# 1,1-Dichlorethen: MAK-Begründung, Nachtrag

**DOI:** 10.34865/mb7535d10_2ad

**Published:** 2025-06-30

**Authors:** Andrea Hartwig, MAK Commission

**Affiliations:** 1 Institut für Angewandte Biowissenschaften. Abteilung Lebensmittelchemie und Toxikologie. Karlsruher Institut für Technologie (KIT) Adenauerring 20a, Geb. 50.41 76131 Karlsruhe Deutschland; 2 Ständige Senatskommission zur Prüfung gesundheitsschädlicher Arbeitsstoffe. Deutsche Forschungsgemeinschaft, Kennedyallee 40, 53175 Bonn, Deutschland. Weitere Informationen: Ständige Senatskommission zur Prüfung gesundheitsschädlicher Arbeitsstoffe | DFG

**Keywords:** 1,1-Dichlorethen, Leber, Niere, Schilddrüse, Lunge, Kanzerogenität, Genotoxizität, Hautresorption, 1,1-dichloroethene, liver, kidney, thyroid, lung, carcinogenicity, genotoxicity, skin absorption

## Abstract

The German Senate Commission for the Investigation of Health Hazards of Chemi﻿cal Compounds in the Work Area (MAK Commission) summarized and evaluated the data for 1,1-dichloroethene [75-35-4] considering all toxicological end points. Relevant stud﻿ies were identified from a literature search. In two-year carcinogenicity studies, 1,1-﻿dichlor﻿oethene increased the incidences of adenomas of the nasal respiratory epithelium in rats and of rare renal tubular carcinomas in male rats. In female rats, the incidences of C-cell adenomas and carcinomas of the thyroid gland were increased with statistical significance at the lowest concentration tested of 25 ml/m^3^ and above. In male mice, the incidences of renal adenomas and carcinomas were increased with sta﻿tisti﻿cal significance at the lowest concentration tested of 6.25 ml/m^3^ and above; rare hepa﻿tocholangiocarcinomas developed in animals of all exposure groups. The incidences of hepatocellular carcinomas were increased with statistical significance in female mice at 12.5 ml/m^3^ and above. After exposure to 25 ml/m^3^, bronchiolar-alveolar carcinomas, hae﻿mangiosarcomas of the liver and the sum of haemangiomas and sarcomas in all organs in female mice were increased with statistical significance. On the basis of these effects, 1,1-dichloroethene has been classified in Carcinogen Category 2. 1,1-﻿Dichloroethene is mutagenic and clastogenic in vitro after metabolic activation. Genotoxicity was observed in vivo in indicator tests in metabolically competent tissues (lungs, liver, kidneys). However, no clastogenicity was induced in the bone marrow or germ cells, even at high concentrations. Thus, 1,1-dichloroethene has not been classified in a germ cell mutagenicity category. However, a genotoxic mechanism of action for the carcinogenic effects cannot be ruled out. Developmental toxicity in rats and rabbits such as variations, delayed ossification (rats) and increased resorptions (rabbits) occurred only with concomitant maternal toxicity in the form of reduced body weight gains. In vitro studies found that 1,1-dichloroethene is absorbed via the skin. The substance has been designated with “H” because of the systemic carcinogenic effects. A sensitizing potential is not expected based on the data available.

**Table:** 

**MAK-Wert**	**–**
**Spitzenbegrenzung**	**–**
	
**Hautresorption (2024)**	**H**
**Sensibilisierende Wirkung**	**–**
**Krebserzeugende Wirkung (2024)**	**Kategorie 2**
**Fruchtschädigende Wirkung**	**–**
**Keimzellmutagene Wirkung**	**–**
	
	
Synonyma	asymmetrisches Dichlorethen 1,1-Dichlorethylen Vinylidenchlorid
Chemische Bezeichnung (IUPAC-Name)	1,1-Dichlorethen
CAS-Nr.	75-35-4
Formel	Cl_2_C=CH_2_
	C_2_H_2_Cl_2_
Molmasse	96,94 g/mol
Schmelzpunkt	–122 °C (IFA 2023)
Siedepunkt bei 1013 hPa	32 °C (ATSDR 2022)
Dichte bei 20 °C	1,25 g/cm^3^ (IFA 2023)
Dampfdruck bei 20 °C	667 hPa (IFA 2023)
log K_OW_	2,13 (IFA 2023)
Löslichkeit bei 25 °C	3,4 g/l Wasser (IFA 2023)
**1 ml/m^3^ (ppm) ≙ 4,022 mg/m^3^**	**1 mg/m^3^ ≙ 0,249 ml/m^3^ (ppm)**
	
Hydrolysestabilität	stabil in Wasser (ECHA 2023)
Verwendung	Intermediat in der Plastikherstellung (ATSDR 2022)

Zu 1,1-Dichlorethen liegen eine Begründung (Henschler 1979), ein umfassender Nachtrag (Henschler 1986) und ein Nachtrag zur Spitzenbegrenzung (Greim 2001) vor. Da seitdem neue Daten veröffentlicht wurden, erfolgt eine Neubewertung aller Endpunkte. Der Nachtrag stützt sich u. a. auf Zusammenfassungen von ATSDR (2022), NTP (2015), IARC (2019) und US EPA (2002).

## 1 Allgemeiner Wirkungscharakter

1,1-Dichlorethen verursacht adverse Effekte in Organen, in denen es metabolisch zu 1,1-Dichlorethenepoxid, 2,2-﻿Dichlor﻿acetaldehyd und 2-Chloracetylchlorid aktiviert wird (Nase, Leber, Nieren und Lunge). Nach inhalativer Exposition treten chronische Entzündungen, Fibrosen und Nekrosen in Leber und Nieren von Mäusen und Ratten, sowie Schädigungen der Keulenzellen in der Lunge von Mäusen und in der Nase von Ratten auf. Nach oraler Verabreichung betrifft die Toxizität im Nager vor allem Leber und Nieren.

1,1-Dichlorethen wirkt bei Ratten und Mäusen kanzerogen; nach 2-jähriger inhalativer Exposition treten bei Ratten in der Nase Adenome des respiratorischen Epithels auf. Bei weiblichen Ratten sind die Inzidenzen für C-Zelladenome und -karzinome der Schilddrüse erhöht. Bei männlichen Ratten sind konzentrationsunabhängig seltene renale Tubuluskarzinome zu beobachten. Bei den männlichen Mäusen kommt es zu Nierenadenomen und -karzinomen, bei weiblichen Mäusen zu hepatozellulären Karzinomen, bronchiolo-alveolären Karzinomen, Hämangiosarkomen der Leber sowie Hämangiomen und -sarkomen (kombiniert) in verschiedenen Organen. Bei beiden Geschlechtern ist die Inzidenz der seltenen Hepatocholangiokarzinome erhöht. Die Tumoren treten ab den niedrigsten getesteten Konzentrationen von 25 ml/m^3^ bei Ratten und 6,25 ml/m^3^ bei Mäusen auf.

1,1-Dichlorethen ist nach metabolischer Aktivierung in vitro mutagen und klastogen. Indikatortests in metabolisch kompetenten Geweben, die die Zielgewebe der Kanzerogenität darstellen, weisen auf eine genotoxische Wirkung hin. Aufgrund der negativen Ergebnisse der Tests im Knochenmark und in den Keimzellen ist zu vermuten, dass die reaktiven Metaboliten lokal begrenzt reagieren.

Entwicklungstoxische Effekte bei Ratten und Kaninchen wie Variationen, Ossifikationsverzögerungen und erhöhte Resorptionen treten nur bei gleichzeitiger Maternaltoxizität in Form von verringerter Körpergewichtszunahme auf.

In einer In-vitro-Untersuchung mit rekonstituierter humaner Epidermis wirkt 1,1-Dichlorethen nicht ätzend. An der Rindercornea in vitro ist der Stoff mäßig augenreizend.

Es liegen keine Untersuchungen zu einer sensibilisierenden Wirkung beim Menschen vor. Tierexperimentelle Untersuchungen sowie Untersuchungen aus tierversuchsfreien Alternativverfahren (NAMs) liefern keine Hinweise auf ein sensibilisierendes Potenzial. Zur atemwegssensibilisierenden Wirkung liegen keine Daten vor.

## 2 Wirkungsmechanismus

### 2.1 Kanzerogenität

1,1-Dichlorethen wird metabolisch vor allem durch CYP2E1 überwiegend zu 1,1-Dichlorethenepoxid oxidiert und in Folgereaktionen zu 2-Chloracetylchlorid und 2,2-Dichloracetaldehyd umgewandelt, welche mit Glutathion (GSH) oder Cystein konjugiert, an Makromoleküle binden oder hydrolysiert werden können (ATSDR 2022; US EPA 2002). Die Bildung des Epoxids und die Konjugation erfolgt vor allem in den zentrilobulären Hepatozyten und Keulenzellen der Lu﻿n﻿ge, in denen CYP2E1 stark exprimiert ist (Forkert 2001). Die in der Leber gebildeten GSH-Konjugate oder ihre Folgeprodukte könnten in den Nieren durch die β-Lyase gespalten und z. B. aus *S*-(2,2-Dichlor-1-hydroxy)ethylglutathion das reaktive Dichlorthioketen gebildet werden (US EPA 2002). In den Nieren können die Cystein-Konjugate auch durch eine renale *S*-Oxidase gespalten werden. Dafür spricht, dass die adverse Wirkung von 1,1-Dichlorethen auf die Nierentubuli von Mäusen um 50 bzw. 60 % reduziert wurde, wenn die Tiere mit Inhibitoren der β-Lyase (Aminooxyessigsäure) oder der *S*-Oxidase (Methimazol) vorbehandelt wurden. Dagegen war die Hemmung der Gamma-Glutamyltranspeptidase und der organischen Aniontransporter ohne Einfluss auf die Toxizität (Ban et al. 1995).

Die Menge an Zellschäden und kovalent gebundenen Metaboliten korreliert in Nieren, Lunge und Leber mit der Konzentration an CYP2E1 in bestimmten Zellen dieser Gewebe (z. B. Lunge: Keulenzellen) und dem Gehalt an GSH (ATSDR 2022; Forkert 2001; US EPA 2002). Ist der GSH-Pool erschöpft, kommt es zu Zellschädigungen. Folglich wird auch die toxische Wirkung durch Fasten der Tiere, durch das es zu einer Verminderung des freien GSH-Gehaltes kommt, verstärkt (ATSDR 2022). Verringert wird die Toxizität durch Substanzen, die den Metabolismus von CYP allgemein oder CYP2E1 im Speziellen herabsetzen oder durch die vermehrte Bildung von intrazellulärem GSH, z. B. bei Hypothyreose (US EPA 2002).

Auch nach oraler Gabe von 1,1-Dichlorethen an Mäuse kommt es zu Schäden in der Lunge, wenn die Dosis so hoch ist, dass die Menge an gebildeten Metaboliten in der Leber nicht vollständig entgiftet werden kann oder systemisch verfügbares 1,1-Dichlorethen direkt in Keulenzellen der Lunge aktiviert wird (US EPA 2002).

Die spezies- und geschlechtsspezifische nephrotoxische Wirkung von 1,1-Dichlorethen wurde in Nierenmikrosomen von Swiss-Webster-Mäusen anhand der Oxidation von 1,1-Dichlorethen in Abhängigkeit des Hormonstatus untersucht. Wurden die Tiere vorher kastriert war die Oxidationsrate vermindert, war aber durch Gabe von Testosteron reversibel. In Nierenmikrosomen der unbehandelten weiblichen Maus war die Oxidationsrate signifikant geringer als die der männlichen Tiere, stieg jedoch deutlich bei Gabe von Testosteron an (Speerschneider und Dekant 1995). Die Expression von CYP2E1 in den Mausnieren wird von Testosteron reguliert (Sohn et al. 2001).

CYP2E1 wird auch in der Nase von Nagern exprimiert (Hartwig und MAK Commission 2022). Dadurch kommt es in der Nase von F344-Ratten zu Zellschäden, Inflammation und in Folge der toxischen Wirkung bei männlichen Tieren zu Adenomen im respiratorischen Epithel (siehe Abschnitt 5.7.2; NTP 2015). Ähnliche adverse Effekte in der Rattennase wurden auch nach Exposition gegen Chloroform nachgewiesen, welches ebenfalls durch CYP2E1 aktiviert wird und die Rattennase schädigt (Hartwig und MAK Commission 2022). Für das humane Nasengewebe liegen bisher keine Untersuchungen vor, die eine Expression von CYP2E1 auf Proteinebene nachweisen. Auf Transkriptionsebene wurde mittels qualitativer Polymerase Chain Reaction in der Nasenschleimhaut humaner Feten *CYP2E1* identifiziert (Zhang et al. 2005). In der humanen Nasenschleimhaut von Erwachsenen wurden *CYP2A6*, *CYP2A13*, CYP2C und CYP3A, nicht aber CYP2E1 detektiert (Ding und Kaminsky 2003). Somit ist wahrscheinlich der Mensch, aufgrund sehr geringer bis fehlender Gehalte an CYP2E1 in der Nase, für die beschriebenen Effekte unempfindlicher als die Ratte (Hartwig und MAK Commission 2022). Vergleichende Daten zur Aktivierung von 1,1-Dichlorethen in der Nase von Ratte und Mensch liegen jedoch nicht vor. Studienresultate mit humanen Lungenmikrosomen legen nahe, dass auch andere CYP-Isoenzy﻿me an der metabolischen Aktivierung von 1,1-Dichlorethen beteiligt sind (Dowsley et al. 1999).

In den Langzeitstudien traten erhöhte Inzidenzen an renalen Tubulusadenomen und -karzinomen bei der männlichen Maus auf. Diese sind vermutlich auf die Organtoxizität mit anschließender Zellproliferation oder auf eine Aktivierung von Cysteinkonjugaten via β-Lyase in den Nieren und ihren Folgeeffekten zurückzuführen (NTP 2015). Untersuchungen zu Tetrachlorethen konnten aufzeigen, dass der Mensch für eine Aktivierung von Cysteinkonjugaten via β-Lyase in den Nieren unempfindlicher ist als der Nager (Hartwig und MAK Commission 2017). Abschätzungen für 1,1-Dichlorethen liegen nicht vor.

### 2.2 Mesotheliome und mononukleäre Zellleukämien

In Langzeituntersuchungen des NTP entwickelten sich nach inhalativer Exposition gegen 1,1-Dichlorethen Mesotheli﻿o﻿me bei männlichen F344-Ratten. Eine Genexpressionsanalyse der Mesotheliome zeigte eine verstärkte DNA-Schädigung und -Reparatur von Zellsignalwegen, die mit Immundysfunktion und Proinflammation in Zusammenhang stehen. Die beschriebenen Mesotheliome gehen in beiden Fällen von der Tunica vaginalis aus (NTP 2015). Derartige Neoplasien bei F344-Ratten werden als stammspezifisch angesehen und auf Störungen im endokrinen System mit nachfolgendem hormonellen Ungleichgewicht zurückgeführt (Maronpot et al. 2009). Auch die mononukleären Zellleukämien bei F344-Ratten sind spezifisch für diesen Tierstamm (Laube et al. 2019).

### 2.3 Genexpressions- und Mutationsanalysen

Globale Genexpressionsprofile zeigten sowohl in nicht-transformiertem Nierengewebe als auch in Nierenzellkarzinomen von männlichen B6C3F1-Mäusen, die gegen 6,25; 12,5 oder 25 ml 1,1-Dichlorethen/m^3^ exponiert waren, eine Überrepräsentation von Genkategorien, die in Signalwegen für oxidativen Stress (z. B. Nuclear factor erythroid 2-related factor 2; Nrf2), Fremdstoffmetabolismus (GSH-Stoffwechsel), Zellzykluskontrolle, Reparatur von DNA-Schäden oder Zellwachstum und -proliferation eine Rolle spielen. In Nierenzellkarzinomen waren zusätzlich c-Myc und P53-Signaling fehlreguliert (Hayes et al. 2016). Da eine Genexpressionsanalyse nur eine Momentaufnahme darstellt, ist aus diesen Daten kein kausaler Zusammenhang für die Kanzerogenese ableitbar. Aufschlussreich wären zusätzliche Untersuchungen zu früheren Zeitpunkten bei verschiedenen Konzentrationen.

Eine Analyse der Mutationen in den Tumoren der B6C3F1-Mäuse zeigte in Lunge und Leber im Vergleich zu spontan aufgetretenen Tumoren keine erhöhten Mutationshäufigkeiten. In den Nierenzellkarzinomen war die Mutationshäufigkeit etwa achtfach erhöht, jedoch lagen keine spontan aufgetretenen Tumore von Kontrolltieren zum Vergleich vor. Die extrahierten Mutationssignaturen zeigten, dass eine neue, vorher noch nicht beschriebene Mutationssignatur in allen drei Tumortypen auftrat und in den Nieren die meisten akkumulierten Mutationen auf diesen mutagenen Prozess zurückgehen. Die Signatur ist gekennzeichnet durch T > A- und T > C-Substitutionen und einem „Strand-Bias“ mit mehr Mutationen auf dem nicht-transkribierten DNA-Strang. Die Mutationssignatur für reaktive Sauerstoffspezies war zwar in allen drei Tumortypen detektierbar, jedoch waren weniger als 10 % der akkumulierten Mutationen in den Nieren auf diese zurückzuführen. Die Analyse der Driver-Mutanten zeigte Mutationen in Genen für Zellwachstum in Lungenkarzinomen (*Braf, Fgfr2, Kras*) und Leberkarzinomen (*Hras*). In den Nierentumoren traten Driver-Mutanten für oxidativen Stress, Tumorpromotion und Apoptose (*Keap1*), Inflammation und Apoptose (*Tnfaip3*) sowie für Invasion und Meta﻿stasierung (*Ddr2*) auf. Diese Driver-Mutationen können teilweise auf die neu entdeckte Mutationssignatur, sowie auf unspezifische mutagene „Alterungs“-Prozesse zurückgeführt werden, die in den meisten humanen Tumoren beobachtet werden (siehe Abschnitt 5.6.2) (Riva et al. 2020). Die Mutation in *Keap1* wäre auch eine Erklärung für die von Hayes et al. (2016) beschriebene aberrante Genexpression des Nrf2-Signalweges, da Keap1 Nrf2 reguliert. Diese Ergebnisse sowie die Daten zur Genotoxizität (siehe Abschnitt 5.6) geben Hinweise auf eine genotoxische Wirkung im Zielgewebe der Kanzerogenität; in welchem Ausmaß diese am Wirkungsmechanismus beteiligt ist, lässt sich derzeit nicht vollständig aufklären.

### 2.4 Fazit

1,1-Dichlorethen wird vor allem durch CYP2E1 zu reaktiven Intermediaten metabolisiert, welche durch GSH entgiftet werden oder an Makromoleküle binden können. In den metabolisch kompetenten Geweben von Ratten und Mäusen treten in Abhängigkeit der Menge an freien reaktiven Metaboliten entsprechend Zellschäden, Zellproliferationen, Inflammation und Nekrosen auf, welche als Ursachen für die kanzerogene Wirkung in Nase, Lunge, Nieren, Leber und Schilddrüse zu sehen sind. Möglicherweise ist der Mensch aufgrund geringerer CYP2E1-Expression weniger sensitiv für Tumoren in der Nase. Studienresultate mit humanen Lungenmikrosomen legen nahe, dass auch andere CYP-Isoenzyme an der metabolischen Aktivierung von 1,1-Dichlorethen beteiligt sind. Ebenso ist es möglich, dass der Mensch gegenüber einer adversen Wirkung in den Nieren aufgrund der Aktivierung von Cysteinkonjugaten durch β-Lyase unempfindlicher ist. Jedoch liegen hierzu keine Abschätzungen vor. Die beschriebenen Mesotheliome sowie die mononukleäre Zellleukämie bei F344-Ratten sind spezies- bzw. stammspezifisch und werden daher als nicht humanrelevant angesehen.

## 3 Toxikokinetik und Metabolismus

Die Toxikokinetik und der Metabolismus von 1,1-Dichlorethen sind umfassend an Ratte und Maus untersucht worden. Im Folgenden werden bereits diskutierte (Henschler 1979, 1986) und neue Daten zusammen dargestellt.

### 3.1 Toxikokinetik

Toxikokinetische Untersuchungen am Menschen liegen nicht vor.

#### 3.1.1 Inhalation

Der Blut:Luft-Verteilungskoeffizient von 1,1-Dichlorethen bei Ratten beträgt 5 (D’Souza und Andersen 1988).

Nach inhalativer Exposition erfolgte die Aufnahme von 1,1-Dichlorethen bei Sprague-Dawley-Ratten bis 150 ml/m^3^ linear. Oberhalb dieser Konzentration sank die prozentuale Aufnahme mit zunehmender Konzentration ab (Dallas et al. 1983; McKenna et al. 1978 b). 1,1-Dichlorethen wurde im Blut von Ratten dieses Stammes bereits zwei Minuten nach Expositionsbeginn detektiert (Dallas et al. 1983).

Nach statischer Ganzkörperexposition von Holtzmann-Ratten (Anfangskonzentration im Exsikkator 2000 ml ^14^C-1,1-Dichlorethen/m^3^, zwei Stunden; Jaeger et al. 1977) oder Sprague-Dawley-Ratten (10 oder 200 ml ^14^C-1,1-Dichlorethen/m^3 ^für sechs Stunden, Untersuchung 72 Stunden nach Expositionsende) (McKenna et al. 1977, 1978 b) wurde der größte Anteil der Radioaktivität in Leber und Nieren (Lunge und Haut nicht untersucht; Jaeger et al. 1977) bzw. Leber, Nieren, Lunge und Haut detektiert. Der Anteil kovalent gebundener Radioaktivität war in der Leber der Tiere am größten, bei nüchternen Ratten mehr als bei nicht gefasteten Tieren (McKenna et al. 1977, 1978 b).

Nach inhalativer Aufnahme wurde 1,1-Dichlorethen schnell metabolisiert und von Sprague-Dawley-Ratten größtenteils mit dem Urin ausgeschieden. Bei einer sechsstündigen Exposition gegen 10 ml/m^3^ waren es innerhalb von drei Tagen nach Expositionsende 74–78 %, bei 200 ml/m^3^ 70–75 % und als CO_2_ abgeatmet wurden 8,7 % bzw. 8,2 % (McKenna et al. 1978 b). Bei dreistündiger Exposition anästhesierter Ratten gegen 25–150 ml 1,1-Dichlorethen/m^3^ (intratracheal mittels eines Atmungsventils) wurden innerhalb von 30 Minuten Gleichgewichtskonzentrationen an 1,1-Dichlorethen in der Ausatemluft erhalten (72–77 % Resorption). Bei 300 ml/m^3^ zeigten die Messungen der Ausatemluft eine Sättigung der Aufnahme an (< 60 % Resorption) (Dallas et al. 1983). Die Kinetik der pulmonalen Abatmung verlief bei Sprague-Daw﻿ley-Ratten biphasisch. Nach Exposition gegen 10 und 200 ml/m^3^ betrugen die Halbwertszeiten für die erste und zweite Phase, basierend auf unverändert abgeatmeter Ausgangssubstanz, 20 und 217 Minuten bzw. 21 und 133 Minuten. Die Kinetik der Ausscheidung mit dem Urin folgte einem ähnlichen Verlauf. Hierbei betrugen die Halbwertszeiten der ersten und zweiten Phase, basierend auf der Gesamtradioaktivität im Urin, 3,1 und 19,3 Stunden bzw. 3,8 und 23,9 Stunden. Der Hauptanteil der Ausscheidung nach Exposition erfolgte in der ersten Phase. Dies gilt sowohl für die Abatmung als auch die Ausscheidung mit dem Urin. Der Ernährungszustand der Ratten beeinflusste die Kinetik der Ausscheidung nicht (McKenna et al. 1977, 1978 b).

Untersuchungen zur Toxikokinetik von 1,1-Dichlorethen im Plasma von Sprague-Dawley-Ratten (keine Futtergabe 24 Stunden vor Exposition) ergaben nach Exposition mittels Atemmaske gegen 100 oder 300 ml/m^3^ (zwei Stunden, Ein- und Ausatemluft getrennt geführt) C_max_-Werte von 0,6 bzw. 2,8 mg/l und die Werte der AUC_0–∞_ betrugen 72 bzw. 279 µg × min/ml. Die Halbwertszeit der Ausscheidung war 50 Minuten. Die Bioverfügbarkeit bei der höchsten Konzentration betrug 55,7 % (Bruckner et al. 2010).

Zur Toxikokinetik bei Mäusen liegen nur wenige Daten vor. Nach sechsstündiger Exposition gegen 10 ml ^14^C-1,1-Dichlorethen/m^3^ wurde bei Ha-ICR-Mäusen ein größerer Anteil im Körper verbleibender Radioaktivität im Vergleich zu gleichbehandelten Ratten festgestellt (Mäuse 5,3 mg-eq ^14^C-1,1-Dichlorethen, Ratten 2,89 mg-eq ^14^C-1,1-Dichlorethen). Die gebundene Radioaktivität in der Leber und den Nieren der Mäuse war ebenfalls größer als in den entsprechenden Organen der Ratten. Die Mäuse atmeten bei dieser Konzentration einen geringeren Anteil an Ausgangssubstanz ab (Mäuse 0,65 %, Ratte 1,63 %) und die Ausscheidung mit dem Urin war größer (Mäuse 80,8 %, Ratten 74,7 %). Dies weist darauf hin, dass Ha-ICR-Mäuse 1,1-Dichlorethen intensiver verstoffwechseln als Sprague-Dawley-Ratten (McKenna et al. 1977).

#### 3.1.2 Orale Aufnahme

Nach Gabe von 0,5–100 mg/kg KG via Schlundsonde wurde 1,1-Dichlorethen schnell und nahezu vollständig von Spra﻿gue-Dawley-Ratten sowie Alderley-Park-Ratten und Alderley-Park-Mäusen aufgenommen und in alle untersuchten Gewebe verteilt (Chieco et al. 1981; Jones und Hathway 1978 b; McKenna et al. 1978 a; Putcha et al. 1986; Reichert et al. 1979), wobei sich der größte Anteil 72 Stunden nach Schlundsondengabe in Leber und Nieren fand (McKenna et al. 1978 a). Die schnelle und vollständige orale Aufnahme wurde im Vergleich zu Ergebnissen nach intravenöser Gabe geschlussfolgert. Die höchsten Konzentrationen im Blut nach oraler Gabe wurden bereits nach 2–8 Minuten festgestellt (Putcha et al. 1986). Die Kinetik der Ausscheidung war ähnlich wie nach inhalativer Exposition. Nach einmaliger oraler Gabe von 1 mg/kg KG wurden von Ratten ca. 3 % der Dosis innerhalb von 72 Stunden als unveränderte Ausgangssubstanz abgeatmet, etwa 11 % als CO_2_, 63 % mit dem Urin und 16 % mit den Faeces ausgeschieden. Bei Gabe von 50 mg/kg KG wurden ca. 19 % als Ausgangssubstanz abgeatmet und 4 % als CO_2_. Mit dem Urin wurden bei dieser Dosis 47 % der applizierten Radioaktivität ausgeschieden. Die Daten lassen auf eine Sättigung des Metabolismus bei relativ geringen Dosisgaben schließen (Jones und Hathway 1978 a; McKenna et al. 1978 a; Reichert et al. 1979).

Der Ernährungszustand der Ratten beeinflusste in geringer Weise die Ausscheidung: es wurden nach einer Gabe von 50 mg/kg KG 29 % als unveränderte Ausgangssubstanz abgeatmet und bei nüchternen Ratten (keine Futtergabe 18 Stunden vor Exposition) 19 % (McKenna et al. 1978 a).

Die Halbwertszeiten der biphasischen Ausscheidung betrugen für die pulmonale Abatmung 25 und 117 Minuten bei 1 mg/kg KG und bei 50 mg/kg KG 21 und 66 Minuten. Die Halbwertszeiten der ersten und zweiten Phase für die Ausscheidung mit dem Urin betrugen bei diesen beiden Dosen 6 und 16,8 Stunden (McKenna et al. 1978 a).

Eine Gabe von 10 oder 30 mg/kg KG via Schlundsonde an Sprague-Dawley-Ratten (keine Futtergabe 24 Stunden vor Exposition) führte zu C_max_-Werten von 2,2 bzw. 8,9 mg/l Plasma und die Werte der AUC_0–∞_ betrugen 51 bzw. 233 µg × min/ml. Die Halbwertszeit der Ausscheidung war 47 bzw. 88 Minuten, die Bioverfügbarkeit bei der höchsten Dosis 46,5 %. Diese Werte waren somit ähnlich wie die nach inhalativer Exposition gegen 10 und 30 ml/m^3^ bis auf die drei- bis vierfach höhere C_max_ (Abschnitt 3.1.1). Bei intragastraler Infusion mit denselben Dosen über zwei Stunden war C_max_ deutlich geringer, die AUC bis zwei Stunden p. a. ebenfalls. Die Kinetik hatte Charakteristika einer First-Pass-Elimination. Die Verteilung aus dem Blut in die Gewebe verlief schneller als die Rückverteilung (Bruckner et al. 2010).

##### 3.1.2.1 Intraperitoneale Aufnahme

Männliche adulte C57Bl/6N-Mäuse (Gewicht: 20–25 g) erhielten einmalig eine intraperitoneale Injektion von 125 mg ^14^C-1,1-Dichlorethen/kg KG in einer Mischung aus Oliven- und Mineralöl (Gesamtvolumen 0,1 ml). Die Verteilung der Radioaktivität in den Organen wurde sechs Stunden bis vier Tage nach Dosisgabe untersucht. Die höchste Menge an Radioaktivität wurde zuerst in der Leber (sechs Stunden nach Dosisgabe) und danach in den Nieren (zwölf Stunden nach Dosisgabe) festgestellt. Zudem wurde im Lungengewebe mehr Radioaktivität detektiert als in Gehirn, Herz oder Muskelgewebe. Eine kovalente Bindung der Radioaktivität war in den Nieren stärker ausgeprägt als in der Leber oder der Lunge und erreichte ihre höchsten Werte im Zeitraum von 6–12 Stunden nach Dosisgabe. In den Nieren lag ab zwölf Stunden bis zur letzten Untersuchung nach vier Tagen nur noch kovalent gebundene Radioaktivität vor. In Leber, Nieren und Lunge war die kovalent gebundene Radioaktivität gleichmäßig über alle subzellulären Fraktionen verteilt, mit einem leicht erhöhten Anteil in der Mitochondrien-Fraktion (Okine et al. 1985) (siehe Abschnitt 2 und 3.2).

#### 3.1.3 Dermale Aufnahme

Aufgrund der physiko-chemischen Eigenschaften von 1,1-Dichlorethen (geringe Größe, Fettlöslichkeit) ist eine Aufnahme der Substanz über die Haut anzunehmen. Es ist jedoch auch von einer hohen Verflüchtigung des Stoffes bei Hautauftragung aufgrund seines hohen Dampfdrucks auszugehen (ATSDR 2022).

In einer In-vitro-Untersuchung wurden der Permeabilitätskoeffizient K_p_ und die Fluxraten nach 10 und 60 Minuten an menschlicher Bauchhaut (entweder dermatomierte oder durch Hitze separierte epidermale Membranen von mindestens sechs Stellen und von mindestens drei Personen) bestimmt. Radioaktiv markiertes 1,1-Dichlorethen wurde unverdünnt in statischen oder Durchflusskammern (k. w. A.) auf die Hautproben okklusiv appliziert. Für die Bestimmung des Permeabilitätskoeffizienten wurden 1200 µl/cm^2^ und für die der Fluxraten nach 10 oder 60 Minuten jeweils 30 µl/﻿cm^2^ aufgetragen. Die Fluxraten nach 10 und 60 Minuten betrugen 800,7 bzw. 144,7 µg/cm^2^ und Stunde und der Permeabilitätskoeffizient nach zwei Stunden betrug 2173 cm/h. Da die Versuche bei 32 °C durchgeführt wurden, war die Wiederfindung in den Experimenten reduziert (73–80 %). Dies ist mit der Volatilität von 1,1-Dichlorethen zu erklären. Nach Berechnungen der Autoren würde die bei einem Arbeitsplatzgrenzwert von 5 ml/m^3^ für einen 8-Stunden-Arbeitstag erreichte innere Belastung via Inhalation bei alleiniger dermaler Exposition über beide Hände nach etwa sieben Minuten erreicht (Fasa﻿no und McDougal 2008). Eine einstündige Exposition beider Hände und Unterarme (2000 cm²) gegen 1,1-﻿Dichlorethen würde bei einem Flux von 144,7 µg/cm² und Stunde zu einer Substanzaufnahme von 289 mg führen. Die inhalativ bei Exposition in Höhe des ehemaligen MAK-Wertes (8 mg/m³, 100 % Resorption, 10 m^3^) über acht Stunden aufgenommene Menge (80 mg) würde bei dermaler Exposition unter den zuvor genannten Bedingungen (144,7 µg/﻿cm²/﻿h, 2000 cm²) innerhalb von ca. 17 Minuten erreicht werden. In der Gesamtschau belegen die In-vitro-Daten eine gute dermale Aufnahme von 1,1-Dichlorethen.

#### 3.1.4 PBPK-Modellierung

Zur Toxikokinetik von 1,1-Dichlorethen wurde ein physiologisch-basiertes pharmakokinetisches (PBPK)-Modell für Ratten nach inhalativer Exposition, oraler Gabe oder intravenöser Gabe entwickelt. Hierbei wurde ein Blut:Luft-Verteilungskoeffizient von 5 gemessen und das Modell anhand mehrerer inhalativer und oraler Toxikokinetik-Tierstudien validiert. Das Modell wurde mit allometrischer Skalierung auf den Menschen übertragen. Das Modell sagte vorher, dass sowohl beim Menschen als auch bei der Ratte der Metabolismus ab etwa 10 mg/kg KG und ab 200 ml/m^3^ gesättigt ist und dass bei gleicher oraler Dosis oder gleicher inhalativer Konzentration an 1,1-Dichlorethen vom Menschen weniger Epoxid gebildet wird als von der Ratte (D’Souza und Andersen 1988). Da das Modell nicht für den Menschen validiert wurde, ist es nicht für eine Risikobewertung für den Menschen nutzbar.

#### 3.1.5 Fazit

1,1-Dichlorethen wird als kleines, hydrophobes Molekül von Ratten und Mäusen rasch nach Inhalation und oraler Gabe ins Blut aufgenommen. In-vitro-Daten belegen zudem eine gute dermale Aufnahme. Die Verteilung erfolgt in alle Gewe﻿be mit höchsten Konzentrationen in Leber und Nieren. Unverändertes 1,1-Dichlorethen wird vor allem abgeatmet; nach oraler Gabe von 50 mg/kg KG an Ratten wurden 28 % als unverändertes 1,1-Dichlorethen abgeatmet und der übrige Anteil schnell weiter metabolisiert (Jones und Hathway 1978 a). Die Elimination bei Ratten erfolgt nach inhalativer und oraler Exposition biphasisch, wobei die größte Menge mit dem Urin (ca. 70 % nach sechs Stunden Exposition gegen 10 oder 200 ml/m^3^) und ein geringerer Teil als CO_2_ (ca. 8 % nach sechs Stunden Exposition gegen 10 oder 200 ml/m^3^) ausgeschieden werden. Die Halbwertszeiten der Ausgangssubstanz für die erste und zweite Phase betrugen 20 bzw. 217 Minuten nach Exposition gegen 10 ml/m^3^ und 21 bzw. 133 Minuten nach Exposition gegen 200 ml/m^3^. Die Halbwertszeiten der ersten und zweiten Phase, basierend auf Gesamtradioaktivität im Urin, betrugen 3,1 bzw. 19,3 Stunden nach Exposition gegen 10 ml/m^3^ und 3,8 bzw. 23,9 Stunden nach Exposition gegen 200 ml/m^3 ^(McKenna et al. 1978 b). Ab 200 ml 1,1-Dichlorethen/m^3^ und oralen Dosen von 50 mg 1,1-Dichlorethen/kg KG ist der Metabolismus bei Ratten gesättigt und nicht metabolisiertes 1,1-Dichlorethen wird abgeatmet. Dies wird durch die lipophilen Eigenschaften der Substanz und ihren kleinen Blut:Luft-Verteilungskoeffizient von 5 (D’Souza und Andersen 1988) begünstigt. 1,1-Dichlorethen wird von Mäusen intensiver als von Ratten metabolisiert.

### 3.2 Metabolismus

Der Metabolismus von 1,1-Dichlorethen wurde insbesondere nach oraler Gabe an Nagern intensiv untersucht. Die bereits beschriebenen Daten (Henschler 1979, 1986) wurden im Folgenden zusammengefasst und um neue Studien ergänzt.

#### 3.2.1 Mensch

Hierzu lagen bisher keine Daten vor. Folgende Studie ist neu hinzugekommen:

1,1-Dichlorethen wurde mit Mikrosomen aus humanem Lungengewebe (8 Patienten, 6 Frauen, 2 Männer, 56–73 Jahre alt, Raucher oder ehemalige Raucher) oder Lebergewebe (9 Organspender, 4 Frauen, 5 Männer, 1–69 Jahre alt, kein Alkoholabusus, keine Angaben zum Raucherstatus) inkubiert. Die Konjugate 2-(*S*-Glutathionylacetylglutathion und 2-*S*-Glutathionylacetat, die eine vorhergehende Bildung von 1,1-Dichlorethenepoxid via CYP2E1 nachweisen, wurden detektiert. In geringen Mengen wurde auch die Acetalform von 2,2-Dichloracetaldehyd gefunden. Bei Zugabe eines spezifischen Inhibitors von CYP2E1 (Diallylsulfon) konnte die Bildung der Konjugate in den humanen Leberansätzen reduziert werden, was die Oxidation via CYP2E1 belegt. Im Gegensatz hierzu konnte Diallylsulfon die Bildung der Konjugate in den humanen Lungenansätzen weniger stark reduzieren. Somit erfolgt in der humanen Lunge, im Gegensatz zur Leber, die Oxidation zu etwa 50 % durch andere CYP-Isoenzyme (Dowsley et al. 1999).

#### 3.2.2 Tier

**Abb. 1:**
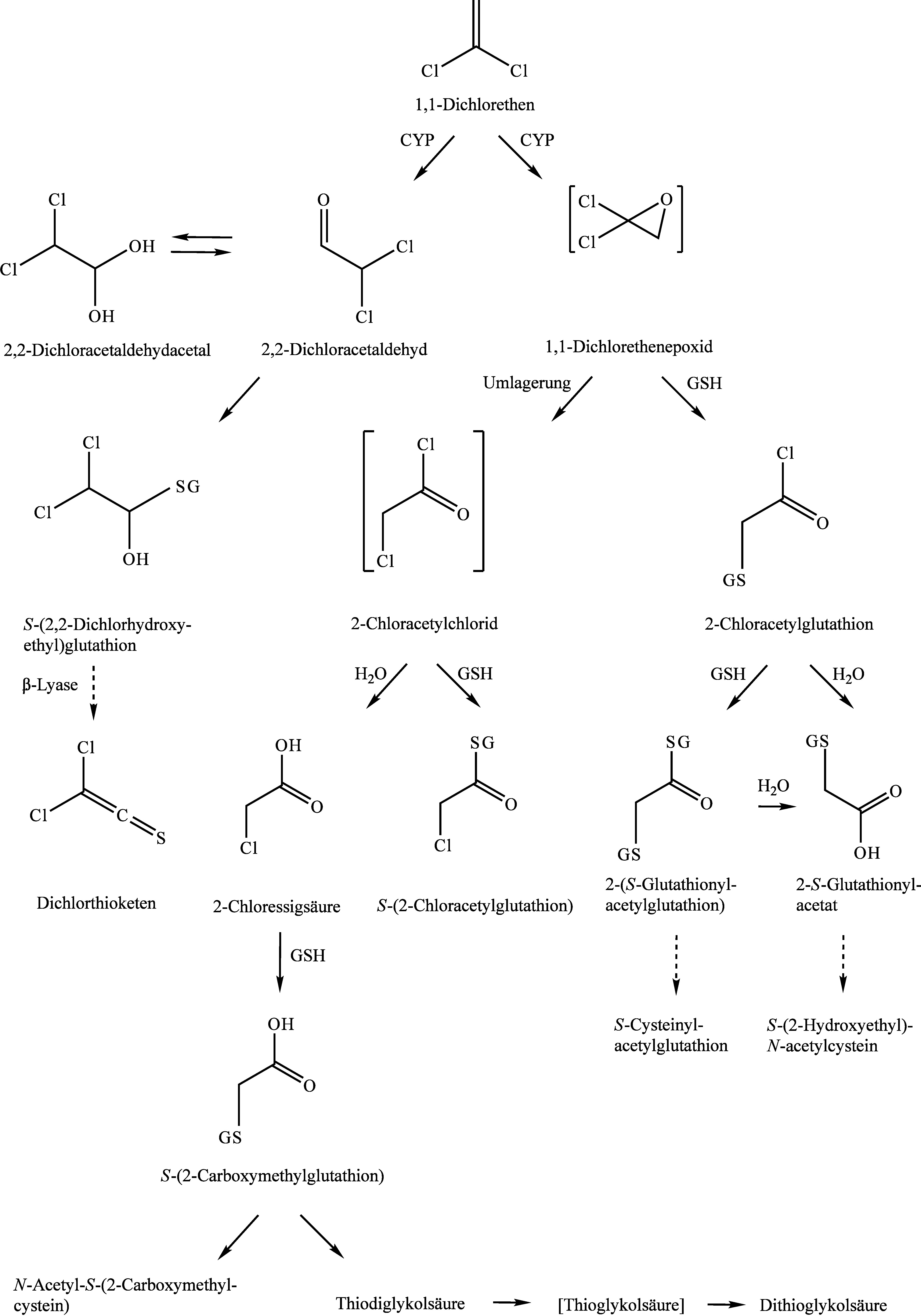
Metabolismusschema von 1,1-Dichlorethen im Nagetier nach ATSDR (2022) und US EPA (2002). Postulierte Metaboliten und Reaktionswege in eckigen Klammern

1,1-Dichlorethen wird durch CYP2E1 zu 1,1-Dichlorethenepoxid oxidiert und es ist anzunehmen, dass sich dieses zu 2-﻿Chloracetylchlorid umlagert; dieser Metabolit wurde jedoch noch nicht nachgewiesen. Zudem wird 1,1-Dichlorethen durch CYP2E1 zu 2,2-Dichloracetaldehyd metabolisiert, das im Gleichgewicht mit seinem Acetal steht, oder mit GSH zu *S*-(2,2-Dichlorhydroxyethyl)glutathion konjugiert wird. Aus diesem könnte in den Nieren durch β-Lyase Dichlorthioketen entstehen (US EPA 2002). Direkt nachgewiesen wurde dies bisher jedoch nicht. Zudem kann der postulierte Metabolit 2-Chloracetylchlorid entweder direkt mit GSH zu *S*-(2-Chloracetylglutathion) konjugiert werden oder hydrolysiert zu 2-Chloressigsäure. Diese wird mit GSH zu *S*-(2-Carboxymethylglutathion) konjugiert und reagiert nach mehreren Schritten zu *N*-Acetyl-*S*-(2-carboxymethylcystein) bzw. zu Thiodiglykolsäure, Thioglykolsäure und Dithiodiglykolsäure. 1,1-Dichlorethenepoxid wird zudem auch in mehreren Konjugationsschritten mit GSH zu *S*-Cysteinylacetylglutathion und *S*-(2-Hydroxyethyl)-*N*-acetylcystein umgewandelt (ATSDR 2022; NTP 2015). Die bedeutende Rolle von Glutathion im Metabolismus wurde durch Studien belegt, die eine Abnahme von GSH-Gehalten der Leber nach Exposition gegen 1,1-Dichlorethen nachwiesen (Jaeger et al. 1974; Reichert et al. 1978; Reynolds et al. 1980). Das Schema des Metabolismus von 1,1-Dichlorethen ist in Abbildung 1 aufgeführt.

#### 3.2.3 Speziesunterschiede 

Mäuse verstoffwechseln einen größeren Anteil 1,1-Dichlorethen nach oraler Gabe als Ratten (siehe Abschnitt 3.1). Dies wurde auch in vitro belegt. Hierbei wurden mit Lebermikrosomen von männlichen CD-1-Mäusen 6-fach höhe﻿re Mengen an Konjugaten, die auf vorhergehender Detoxifizierung des Epoxids basieren, gebildet als von männlichen Spra﻿gue-Dawley-Ratten. Obwohl ansonsten die gleichen Metaboliten bei Mäusen und Ratten entstehen, wur﻿de *N*-﻿Ace﻿tyl-*S*-﻿(2-car﻿boxymethyl)cystein, welches vermutlich aus 2-Chloracetylchlorid gebildet wird, nur bei Mäusen nachgewiesen. Zudem entsteht bei Mäusen mehr *S*-(2-Hydroxyethyl)-*N*-acetylcystein, welches nach Reaktion von 1,1-﻿Di﻿chlo﻿re﻿the﻿ne﻿poxid und GSH gebildet wird. Somit ist anzunehmen, dass Mäuse mehr Epoxid bilden als Ratten (Dowsley et al. 1995). An drei Mausstämmen (A/J-, CD-1- und C57Bl/6-Mäuse) konnte aufgezeigt werden, dass der Stamm mit der höchsten Menge an hepatischem CYP2E1 (A/J) auch am meisten 1,1-Dichlorethenepoxid, kovalent gebundene Intermediate und GSH-Konjugationsprodukte aus dem Epoxid im Leberzytosol bildete. Bei diesem Stamm war auch die 1,1-﻿Dichlorethen-induzierte zentrilobuläre Nekrose in der Leber am höchsten (ATSDR 2022). Zudem wird aufgrund eines Geschlechtsdimorphismus das Enzym CYP2E1 in den Nieren männlicher CD-1- und B6C3F1-Mäuse stärker exprimiert als in weiblichen (Hayes et al. 2016) und die Oxidation von 1,1-Dichlorethen via CYP2E1 wird in den männlichen Mausnieren von Testosteron reguliert (Speerschneider und Dekant 1995). Der Metabolismus ist in den männlichen Mausnieren 6-fach höher als in den weiblichen (IARC 2019). Die Umwandlung von 1,1-Dichlorethenepoxid zu *S*-﻿Cysteiny﻿lacetylglutathion und *S*-(2-Hydroxyethyl)-*N*-acetylcystein ist ein Stoffwechselweg, der bei Mäusen präferiert stattfindet (ATSDR 2022). Zudem wurde das aus 2,2-Dichloracetaldehyd entstehende Acetal mit Lungenmikrosomen der Maus in größerer Menge gebildet als mit Rattenlebermikrosomen. Die Aktivität der β-Thionase ist bei der Maus höher, da mehr Dithioglykolsäure als Thiodiglykolsäure im Metabolismus der Maus nach Exposition gebildet wird (ATSDR 2022; NTP 2015). Da bei Mäusen rasch eine Sättigung der Konjugationsreaktion von Monochloressigsäure und GSH eintritt, reichert sich die Monochloressigsäure an und wird vermehrt mit dem Urin ausgeschieden (Henschler 1979).

#### 3.2.4 Humanrelevanz

In humanen Leber- und Lungenmikrosomen wird 1,1-Dichlorethenepoxid als initialer Metabolit via CYP-Monooxyge﻿nasen gebildet. Im Metabolismus der humanen Leber ist CYP2E1 bei diesem Prozess maßgeblich beteiligt, wohingegen in humanen Lungenmikrosomen vermutlich auch andere CYP-Isoenzyme diesen Schritt katalysieren (Dowsley et al. 1999). Da für die Zytotoxizität das Epoxid als relevantester Metabolit angesehen wird (NTP 2015), sind adverse Folgereaktionen basierend auf Reaktionen dieses elektrophilen Metaboliten mit Zellbestandteilen auch für humane Zellen anzunehmen. Da eine mutagene Wirkung in den Salmonella-Stämmen TA100 und TA1535 in Anwesenheit von humanen Leberzellen (Jones und Hathway 1978 c, siehe Abschnitt 5.6.1) auftritt, werden die für die mutagene Wirkung verantwortlichen Intermediate auch in der humanen Leber gebildet. Das Epoxid wird in humaner Leber und Lunge mit GSH konjugiert. Somit ist auch beim Menschen GSH bei der Entgiftung von 1,1-Dichlorethen bedeutend. Es ist daher anzunehmen, dass der Metabolismus von 1,1-Dichlorethen beim Menschen und beim Nagetier qualitativ ähnlich verläuft, wobei es quantitative Unterschiede gibt: Die Bildung der auf dem Epoxid beruhenden GSH-Konjugate erfolgte in drei von fünf Inkubationen mit humanen Lebermikrosomen in 2,5–3-fach so hoher Menge wie in den Ansätzen mit Lebermikrosomen von CD-1-Mäusen. Mit den humanen Lungenmikrosomen wurde im Vergleich zu denen der CD-Maus nur etwa 50 % der auf dem Epoxid beruhenden GSH-Konjugate gebildet, jedoch waren die entstandenen Mengen in den humanen Ansätzen sehr variabel (Dowsley et al. 1999). Rekombinantes CYP2E1 der Ratte hat eine höhere Affinität und Umsetzungsrate für 1,1-Dichlorethen als humanes CYP2E1, CYP2F2 der Maus, CYP2F3 der Ziege oder CYP2F4 der Ratte (Simmonds et al. 2004). CYP2E1, das zentrale Enzym der Bioaktivierung von 1,1-Dichlorethen in Nagetieren, kommt beim Menschen hauptsächlich in der Leber vor. Seine Konzentration in der humanen Lunge ist niedrig und variabel (IARC 2019). Dies wird primär mit der geringen Anzahl von metabolisch kompetenten Zellen, den Keulenzellen, in diesem Gewebe erklärt (Forkert 2001). In humanen Nierenmikrosomen wurde CYP2E1 gar nicht oder nur in sehr geringer Menge nachgewiesen; CYP2F2 kommt in der Mauslunge häufiger vor als in der humanen Lunge (IARC 2019). Zudem ist der GSH-Gehalt in der Leber beim Menschen höher als bei Ratten (Mertens et al. 1996).

#### 3.2.5 Fazit

1,1-Dichlorethen wird zu 1,1-Dichlorethenepoxid oxidiert und dieses lagert sich spontan zu 2-Chloracetylchlorid um. Zudem wird 1,1-Dichlorethen via Oxidation zu 2,2-Dichloracetaldehyd metabolisiert. Diese Metaboliten sind reaktiv. Eine nachfolgende Konjugation mit GSH und weitere Abbauschritte (Oxidationen, Hydrolysen) führen zu verschiedenen Endprodukten (*S*-Cysteinylacetylglutathion, *S*-(2-Hydroxyethyl)-*N*-acetylcystein, *S*-(2,2-Dichlorhydroxyethyl)glutathion, *S*-(2-Chloracetyl)glutathion, *N*-Acetyl-*S*-(2-carboxymethylcystein) und Dithiodiglykolsäure) (ATSDR 2022; IARC 2019; NTP 2015). *S*-(2,2-Dichlorhydroxyethyl)glutathion könnte in den Nieren durch β-Lyase zu Dichlorthioketen umgewandelt werden (US EPA 2002). Dies wurde jedoch bisher nicht direkt nachgewiesen. Untersuchungen zu Tetrachlorethen konnten aufzeigen, dass der Mensch für eine Aktivierung von Cysteinkonjugaten via β-Lyase in den Nieren unempfindlicher ist als der Nager (Hartwig und MAK Commission 2017). Abschätzungen für 1,1-Dichlorethen liegen nicht vor.

Der Metabolismus bei Ratte und Maus verläuft ähnlich, jedoch ist die Maus bezüglich der Nierentoxizität empfindlicher als die Ratte (ATSDR 2022). Der Metabolismus beim Menschen verläuft initial ähnlich, jedoch scheinen die für die Aktivierung verantwortlichen CYP-Enzyme beim Menschen in der Leber in höherer und in der Lunge in geringerer Aktivität vorzuliegen als beim Nager und die detoxifizierende GSH-Kapazität ist beim Menschen in der Leber höher. Der weitere Metabolismus beim Menschen ist bisher nicht erforscht und die Charakterisierung der beteiligten CYP-Isoenzyme nicht erfolgt.

## 4 Erfahrungen beim Menschen

Die Geruchsschwelle soll etwa bei 50 ml/m^3^ liegen (Henschler 1979).

### 4.1 Einmalige Exposition

Nach akuter Exposition (wenige Minuten, k. w. A.) gegen hohe Luftkonzentrationen von etwa 4000 ml 1,1-﻿Dich﻿l﻿o﻿re﻿then/﻿m^3^ wurde von depressiven Effekten auf das zentrale Nervensystem und Rauschzuständen berichtet, die bis zur Bewusstlosigkeit führen können. Eine vollständige Erholung von diesen Effekten setzte nach Ende der Exposition ein (k. w. A.; ATSDR 2022).

### 4.2 Wiederholte Exposition

Seit dem letzten Nachtrag (Henschler 1986) liegen nur zur entwicklungstoxischen und kanzerogenen Wirkung neue Publikationen vor, die in Abschnitt 4.5 und 4.7 beschrieben werden.

### 4.3 Wirkung auf Haut und Schleimhäute

#### 4.3.1 Haut

Flüssiges 1,1-Dichlorethen wirkt nach wenigen Minuten hautreizend. Ein länger anhaltender Kontakt führt zu Schäden vergleichbar mit Verbrennungen ersten Grades (k. w. A.). Da kommerziellen 1,1-Dichlorethenlösungen Polymerisations-Inhibitoren zugesetzt werden, vor allem *p*-Hydroxyanisol oder Phenol, ist die Hautirritation möglicherweise auf diese Antioxidantien zurückzuführen (ATSDR 2022; US EPA 1979). *p*-Hydroxyanisol führt bei Konzentrationen ab 0,25 % zur Depigmentierung der Haut (ATSDR 2022).

#### 4.3.2 Auge

1,1-Dichlorethen ist augenreizend beim Menschen (k. w. A.; ATSDR 2022).

### 4.4 Allergene Wirkung

Es liegen keine Daten vor.

### 4.5 Reproduktionstoxizität

In einer populationsbasierten Querschnittstudie wurde der Zusammenhang zwischen verunreinigtem Trinkwasser und Befunden bei Neugeborenen in 75 Städten New Jerseys, USA, im Zeitraum von 1985–1988 untersucht. Im Trinkwasser wurden Trichlorethen, 1,1,1-Trichlorethan, 1,2-Dichlorethan, Tetrachlorkohlenstoff, Tetrachlorethen, Benzol und verschiedene Dichlorethene nachgewiesen. Es wurden Assoziationen zwischen der Exposition gegen Gesamt-Dichlorethen und dem Auftreten von Gaumenspalten, von Defekten im zentralen Nervensystem und des Neuralrohrs mit Odds Rati﻿os über 1,5 berichtet (Bove et al. 1995). Ob 1,1-Dichlorethen für die Effekte verantwortlich ist, lässt sich aufgrund der fehlenden Adjustierung für mögliche Risikofaktoren wie berufliche Exposition, Raucherstatus und Alkoholkonsum und der Mischexposition gegen andere Chemikalien nicht abschließend bewerten.

### 4.6 Genotoxizität

Hierzu liegen keine Daten vor.

### 4.7 Kanzerogenität

Aus den bisher vorliegenden Kohortenstudien ließ sich kein erhöhtes Krebsrisiko ableiten (Henschler 1979, 1986).

Eine bisher noch nicht beschriebene retrospektive Kohortenstudie von Waxweiler et al. (1981) untersuchte 4806 männliche Beschäftigte einer Fabrik zur Plastikherstellung in den USA, die gegen 1,1-Dichlorethen, Vinylchlorid, Polyvi﻿nylchlorid-Staub, Acrylate und weitere Chemikalien exponiert waren (Expositionszeitraum 1942–1973). Es wurde eine statistisch signifikant erhöhte Mortalität aufgrund von malignen Neoplasien im Atemtrakt bei den Beschäftigten festgestellt. Die Assoziation der Lungenkrebsfälle mit der Exposition gegen 19 verschiedene Chemikalien, inklusive 1,1-﻿Dichlorethen, wurde basierend auf der Historie der Beschäftigten vorgenommen. Für eine Exposition gegen Polyvinylchlorid-Staub war die Assoziation mit Adenokarzinom der Lunge und großzelligem Lungenkrebs (kombiniert) statistisch signifikant (p = 0,037), wohingegen für 1,1-Dichlorethen die Assoziation statistisch nicht signifikant war (p = 0,267) (Waxweiler et al. 1981). Da die Beschäftigten gegen verschiedene Chemikalien exponiert waren und keine Angaben zur Expositionshöhe vorliegen, wird die Studie bei der Bewertung der Kanzerogenität nicht berücksichtigt.

Aus den vorliegenden epidemiologischen Studien lässt sich für den Menschen keine kanzerogene Wirkung belegen.

## 5 Tierexperimentelle Befunde und In-vitro-Untersuchungen

### 5.1 Akute Toxizität

#### 5.1.1 Inhalative Aufnahme

Nach vierstündiger, inhalativer Exposition betrugen die LC_50_-Werte für normale und gefastete Ratten ab 3800 ml/m^3^ bzw. 2300 ml/m^3^ (Henschler 1979). Die LC_50_-Werte nach 23-stündiger inhalativer Exposition von CD-Mäusen lagen bei 98–105 ml/m^3^ und für gefastete NMRI-Mäuse bei 50–125 ml/m^3^ nach vierstündiger inhalativer Exposition (ATSDR 2022; NTP 2015). Für den Chinesischen Hamster lagen die Werte nach vierstündiger inhalativer Exposition bei 1915–﻿2945 ml/m^3^ und bei 150–﻿455 ml/m^3^ für gefastete Tiere (ATSDR 2022). Nach akuter inhalativer Exposition gegen 180–200 ml/m^3^ wurden im Serum von gefasteten Sprague-Dawley-Ratten erhöhte Aktivitäten von Glutamat-Dehydrogenase, Sorbitdehydrogenase und Alaninaminotransferase und im Urin von Aspartat-Aminotransferase und *N*-Acetylglucosaminidase sowie vermehrt Beta-2-Mikroglobulin als Zeichen von Leber- bzw. Nierenschädigung festgestellt. Bei 150–﻿180 ml/m^3^ waren die Effekte geringer (Cavelier et al. 1996). Vierstündige Expositionen von gefasteten Spra﻿gue-Daw﻿ley-Ratten gegen 250–400 ml/m^3^ resultierten in erhöhten Nierengewichten, Zellschwellungen im renalen Kortex, Serum-Harnstoffstickstoff-Konzentrationen sowie Serum-Kreatiningehalten (Jackson und Conolly 1985). In der Leber traten bei Exposition gegen 200 ml/m^3^ Zellveränderungen im zentrilobulären und midzonalen Bereich sowie Blutungen und Nekrosen im zentrilobulären Bereich und eine Depletion von GSH, sowie eine Zunahme der Transaminasen und von Sorbit-Dehydrogenase im Serum auf (Reynolds et al. 1980).

#### 5.1.2 Orale Aufnahme

Die LD_50_-Werte nach oraler Verabreichung lagen im Bereich von 1500–1800 mg/kg KG für Ratten und bei 194–217 mg/﻿kg KG für Mäuse (NTP 2015).

GSH-Depletion in der Leber wurde bei Wistar-Ratten nach einmaliger oraler Verabreichung von 1000 mg/kg KG (Rei﻿chert et al. 1978) und bei CD-Mäusen nach Verabreichung von 75 mg/kg KG festgestellt (Forkert und Moussa 1991). Ab 400 mg/kg KG waren Harnstoffstickstoff- und Kreatiningehalte sowie relative Nierengewichte bei Sprague-Daw﻿ley-Ratten erhöht (Jenkins und Andersen 1978); bei Mäusen traten ab 125 mg/kg KG Zellschädigungen im midzonalen und zentrilobulären Leberbereich auf (Forkert und Moussa 1991). In Keulenzellen von C57Bl/6-Mäusen kam es zu Zellveränderungen und -schäden (Dilatationen, Degeneration des endoplasmatischen Retikulums) ab 100 mg/kg KG und zu Nekrosen ab 200 mg/kg KG (ATSDR 2022; Forkert und Reynolds 1982).

#### 5.1.3 Dermale Aufnahme

Hierzu liegen keine Daten vor.

#### 5.1.4 Fazit

Mäuse sind gegenüber der toxischen Wirkung nach akuter inhalativer Exposition oder oraler Verabreichung empfindlicher als Ratten. Zielorgane der akuten toxischen Wirkung sind Leber und Nieren von Ratten und Mäusen sowie die Lunge von Mäusen.

### 5.2 Subakute, subchronische und chronische Toxizität

#### 5.2.1 Inhalative Aufnahme

In mehreren älteren Studien mit wiederholter inhalativer Exposition von Ratten und Mäusen sind die Zielorgane der toxischen Wirkung Nieren und Leber. In der Leber treten bei Ratten u. a. zentrilobuläre Hyperplasien, Nekrosen, fettige Veränderungen und Proliferationen der Gallengänge auf, bei Mäusen zentrilobuläre Schwellungen, Hypertrophien und Nekrosen. In den Nieren werden bei Ratten Hypertrophien des Tubulusepithels und bei Mäusen Nephrosen, Entzündungen und erhöhte Nierengewichte festgestellt. Bei Affen, Meerschweinchen und Hunden betrifft die adverse Wirkung die Leber. Hierbei wurden bei Affen und Hunden fettige Veränderungen, fokale Nekrosen, Gallengangsproliferationen und Fibrosen beobachtet, wohingegen bei Meerschweinchen die Aktivität der alkalischen Phosphatase erhöht war. Da es sich dabei um ältere Studien handelt, die in anderen Reviews bereits detaillierter beschrieben sind (ATSDR 2022; US EPA 2002), werden sie hier nicht genauer dargestellt.

In einer bisher noch nicht bewerteten Kanzerogenitätsstudie wurden männliche und weibliche SD-Ratten gegen 75 ml 1,1-Dichlorethen/m^3^ für 18 Monate exponiert. Es wurden leichte fettige Veränderungen der Leber und ein erniedrigtes bzw. erhöhtes Körpergewicht bei weiblichen und männlichen Tieren festgestellt (Quast et al. 1986). In einer weiteren noch nicht bewerteten Kanzerogenitätsstudie kam es nach 104-wöchiger Exposition weiblicher SD-Ratten gegen 100 ml/m^3^ zu vermindertem Körpergewicht (Cotti et al. 1988). Beide Studien sind nur eingeschränkt für eine Bewertung geeignet (dazu und zu neoplastischen Befunden siehe Abschnitt 5.7.2).

In Tabelle 1 sind die nicht-neoplastischen Befunde der NTP (2015)-Studie (siehe Abschnitt 5.7.2) mit ihren Vorstudien aufgeführt, aus denen sich keine NOAEC ergibt. Wie in den älteren Studien sind in diesen Untersuchungen des NTP Leber und Nieren die Zielorgane bei Ratten und Mäusen. Zudem sind weitere Zielorgane die Lunge sowie das Epithel der Nase. In den Langzeitstudien (105 Wochen) betrug die LOAEC für F344-Ratten 25 ml 1,1-Dichlorethen/m^3^. Hierbei wurden renale Tubulushyperplasien (männliche Ratten, nicht statistisch signifikant) sowie Hyperplasien im alveolären Epithel der Lunge (männliche Ratten) und des respiratorischen Epithels (weibliche Ratten) festgestellt. Des Weiteren wurde eine chronische Entzündung, respiratorische Metaplasie, Turbinalien-Atrophie und Hyperostose in der Nase bei beiden Geschlechtern beobachtet. In der Leber männlicher und weiblicher Tiere traten chronische Entzündungen und diffuse fettige Veränderungen auf. Die Bursa ovarica waren dilatiert und es wurden bei weiblichen Tieren auch Fettgewebsnekrosen im Mesenterium festgestellt. Auftretende Mesotheliome verursachten bei männlichen Ratten Noduli im Bauchfell sowie in den Testes und den Epididymides. Die LOAEC für B6C3F1-Mäuse in der Langzeitstudie betrug 6,25 ml 1,1-﻿Dichlorethen/m^3^. Hier wurden bei weiblichen Tieren vermindertes Körpergewicht und abnormale Atmung festgestellt. In der Nase traten bei beiden Geschlechtern bei dieser Konzentration respiratorische Metaplasie, Turbinalien-Atrophie und Hyperostose auf. In den Nieren wurden bei männlichen Mäusen Tubulushyperplasien festgestellt. Im Mesenterium der weiblichen Tiere kam es zu Fettgewebsnekrosen (NTP 2015). Die beschriebenen LOAEC sind niedriger als die bisher erhobenen NOAEC aus älteren Studien (siehe Henschler 1979, 1986).

**Tab. 1:** Wirkung von 1,1-Dichlorethen nach wiederholter inhalativer Verabreichung

**Spezies,** **Stamm,** **Anzahl pro Gruppe**	**Exposition**	**Befunde**	**Literatur**
**Ratte**, F344, je 5 ♂, ♀	**16 Tage,** 6 h/d, 5 d/Wo, 0, 25, 50, 100, 200, 400 ml/m^3^, Ganzkörper	**ab 25 ml/m^3^**: **LOAEC**; Leber: ♂/♀: zentrilobuläre zytoplasmatische Veränderungen in Hepatozyten, ♂: leichte zentrilobuläre Nekrose, Nieren: ♂/♀: abs. u. rel. Gew. ↑;	NTP 2015
		**ab 100 ml/m^3^**: ♀: KG-Zunahme ↓, Lunge: ♂: rel. Gew. ↑; **200 ml/m^3^**: ♂/♀: Lethargie, Mortalität 5/5 ♂ u. 4/5 ♀ bis 2. Tag; **ab 200 ml/m^3^**: Leber: ♂/♀: zentrilobuläre Nekrosen, Hämorrhagie, Nieren: ♂/♀: Harnzylinder in Nierenpapille ↑; **400 ml/m^3^**: ♂/♀: Lethargie u. Ataxie, Mortalität 5/5 ♂ bis 2. Tag u. 5/5 ♀ bis 4. Tag	
**Ratte**, F344, je 10 ♂, ♀	**14 Wochen,** 6 h/d, 5 d/Wo, 0; 6,25; 12,5; 25; 50; 100 ml/m^3^, Ganzkörper	**ab 6,25 ml/m^3^**: **LOAEC**; Nieren: ♂: rel. Gew. ↑, Nase: ♂/♀: Atrophie olfaktorisches Epithel, Mineralisationen, Nekrosen, Atrophie Turbinalien; **ab 12,5 ml/m^3^**: Nieren: ♀: abs. u. rel. Gew. ↑, Leber: ♂: zentrilobuläre zytoplasmatische Veränderungen; **ab 25 ml/m^3^**: ♀: Albuminkonz. ↑; **ab 50 ml/m^3^**: ♂/♀: Harnstoffstickstoff ↑, AP ↑, ♀: SDH ↑, ♂: ALT ↑ (3. Tag), ♀: zytoplasmatische Vakuolisierung, ♂/♀: Gesamtprotein- u. Globulingehalt ↑ (nach 23. Tag vollständig reversibel, Ursache: Dehydrierung); **100 ml/m^3^**: ♂/♀: Hämoglobinkonz. u. Erythrozytenanzahl ↑, Hämatokrit, SDH ↑, ♂: Albuminkonz. ↑, ALT ↑ (23. Tag), Spermienmotilität ↓, abs. u. rel. Spermatidgehalte ↓	NTP 2015
**Ratte**, F344, je 50 ♂, ♀	**105 Wochen,** 6 h/d, 5 d/Wo, 0, 25, 50, 100 ml/m^3^, Ganzkörper	**ab 25 ml/m^3^**: **LOAEC**; Nieren: ♂: renale Tubulushyperplasien (n. s., siehe Abschnitt 5.7.2), Nase: ♂/♀: chron. Entzündungen (inkl. in Atemwegen), Atrophie u. Hyperostose der Turbinalien, resp. Metaplasie olf. Epithel, resp. Epithel: ♀: Hyperplasien, Lunge: ♂: Hyperplasien im alv. Epithel, Leber: ♂/♀: chron. Entzündung, diffuse Fettveränderungen, Ovarien: ♀: Dilatation d. Bursa ovarica, Mesenterium: ♀: Fettnekrosen, Bauchfell: ♂: Noduli (Mesotheliom-bedingt), Testes u. Epididymides: ♂: Noduli (Mesotheliom-bedingt); **ab 50 ml/m^3^**: resp. Epithel: ♂: Hyperplasien, Leber: ♀: Nekrosen, **100 ml/m^3^**: ♀: Mortalität ↑, Leber: ♂/♀: zystische Degeneration, Tumorbefunde u. Vorstufen siehe Tabelle 4	NTP 2015
**Maus**, B6C3F1, je 5 ♂, ♀	**17 Tage,** 6 h/d, 5 d/Wo, 0, 25, 50, 100, 200, 400 ml/m^3^, Ganzkörper	**ab 25 ml/m^3^**: **LOAEC**; ♂: KG-Zunahme ↓, Lunge: ♀: abs. u. rel. Gew. ↑, Leber: ♂/♀: rel. Gew. ↑, Nieren: ♂: renale Tubulusnekrosen, „granuläre Zylinder“; **ab 50 ml/m^3^**: Lethargie 2/5 ♂; abnormale Atmung 1/5 ♂, Leber: ♀: abs. Gew. ↑, Nase: ♂: leichte Nekrosen; **100 ml/m^3^**: Mortalität 5/5 ♂, 4/5 ♀ bis 4. Tag, ♂: Lethargie (5/5), abnormale Atmung (4/5); **ab 100 ml/m^3^**: Leber: ♂/♀: zentrilobuläre Nekrosen, Nase: ♂: Nekrose im resp. Epithel; **ab 200 ml/m^3^**: Mortalität 5/5 ♂/♀ nach 1 Tag, Nase: ♀: Nekrose im resp. Epithel	NTP 2015
**Maus**, B6C3F1, je 10 ♂, ♀	**14 Wochen,** 6 h/d, 5 d/Wo, ♀: 0; 6,25; 12,5; 25; 50; 100 ml/m^3^, ♂: 0; 6,25; 12,5; 25; 50 ml/m^3^, Ganzkörper	**ab 6,25 ml/m^3^**: **LOAEC**; Nieren: ♂: abs. Gew. ↓, Leber: ♀: rel. Gew. ↑; **ab 12,5 ml/m^3^**: ♂/♀: KG-Zunahme, mittleres KG ↓, Blut: ♂: Erythrozytenanzahl ↓, Hämoglobinkonz. ↓, Hämatokritgehalt ↓, Leber: ♀: abs. Gew. ↑, Nieren: ♂: Nephropathie, Testes: ♂: Totalspermienanzahl pro Cauda Epididymis ↓; **ab 25 ml/m^3^**: Hoden: ♂: Cauda Epididymidesgew. ↓; **50 ml/m^3^**: Mortalität 2/10 ♂ bis 7. Tag, Blut: ♀: Erythrozytenanzahl ↓, Hämoglobinkonz. ↓, Hämatokritgehalt ↓, Nieren: ♂: Tubulus-Nekrosen, Proteinzylinder, Kehlkopf: ♂: Plattenepithel-Metaplasie im respiratorischen Epithel; **ab 50 ml/m^3^**: Nieren: ♂: Läsionen, Nekrosen u. Proteinzylinder, Blut: ♀: Erythrozytenanzahl ↓, Leber: ♂: Nekrosen; **100 ml/m^3^**: Mortalität 4/10 bis 7. Tag, Leber: Nekrosen, Hypertrophie zentrilobulärer Hepatozyten, Lunge: abs. u. rel. Gew. ↑, Nekrosen im bronchialen Epithel, histiozytäre Entzündung, Nieren: abs. u. rel. Gew. ↑, Nase: Nekrosen im resp. Epithel u. Atrophie der Turbinalien ↑, Kehlkopf: Hyperplasien im respiratorischen Epithel, Nekrosen, Plattenepithel-Metaplasie	NTP 2015
**Maus**, B6C3F1, je 50 ♂, ♀	**105 Wochen,** 6 h/d, 5 d/Wo, 0; 6,25; 12,5; 25 ml/m^3^, Ganzkörper	**ab 6,25 ml/m^3^**: **LOAEC**; ♀: Mortalität ↑, abnormale Atmung, KG ↓, Abmagerung, Nase: ♂/♀: Atrophie, Hyperostosen der Turbinalien, olf. Epithel: ♂/﻿♀: resp. Metaplasien, Nieren: ♂: Tubulushyperplasien, Mesenterium: ♀: Fettnekrosen, **ab 12,5 ml/m^3^**: ♂: KG-Zunahme > 12 % ↓, olf. Epithel: ♂/♀: hyaline Tröpfchen; **25 ml/m^3^**: ♂: abnormale Atmung, KG ↓, ♀: KG-Zunahme 20 % ↓, Leber: ♂: basophile Foci, Nieren: ♂: Zysten, Tumorbefunde siehe Tabelle 5	NTP 2015

abs.: absolut; ALT: Alaninaminotransferase; AP: alkalische Phosphatase; d: Tag; Gew.: Gewicht; h: Stunde; KG: Körpergewicht; Konz.: Konzentration; n. s.: nicht statistisch signifikant; olf.: olfaktorisch; rel.: relativ; resp.: respiratorisch; SDH: Sorbitdehydrogenase; u.: und; Wo: Woche

#### 5.2.2 Orale Aufnahme

Hierzu liegen keine neuen Studien vor.

Nach oraler Verabreichung sind die Zielorgane vor allem die Leber, Nieren und Lunge bei Ratten sowie die Leber bei Mäusen (ATSDR 2022; Henschler 1986; US EPA 2002). In Langzeitstudien (104 Wochen) des NTP konnten keine NOAEL abgeleitet werden. In diesen Studien wurden bei Verabreichung über die Schlundsonde von 5 mg 1,1-Dichlorethen/kg KG und Tag (LOAEL) an F344-Ratten chronische Entzündungen von Nieren und Lunge (jeweils bei männlichen und weiblichen Tieren) sowie Hyperplasien im Dickdarm von männlichen Tieren festgestellt. Die Verabreichung über die Schlundsonde von 2 mg/kg KG und Tag (LOAEL) für 104 Wochen an B6C3F1-Mäuse führte zu Nekrosen in der Leber und einer erhöhten Mortalität bei männlichen und weiblichen Tieren (NTP 1982). Diese Befunde widersprechen den Ergebnissen einer Studie aus dem Jahr 1983 (Quast et al. 1983) in der ein NOAEL von 7 mg/kg KG und Tag für männliche Sprague-Dawley-Ratten abgeleitet wurde. In dieser Studie wurden bei weiblichen Tieren ab 9 mg/kg KG und Tag midzonale fettige Veränderungen in der Leber und eine Schwellung der Hepatozyten festgestellt. Diese Befunde waren bei männlichen Tieren erst ab einer Dosis von 20 mg/kg KG und Tag statistisch signifikant.

#### 5.2.3 Dermale Aufnahme

Hierzu liegen keine neuen Daten vor.

#### 5.2.4 Fazit

Nach inhalativer Exposition gegen 1,1-Dichlorethen sind die Zielgewebe bei Nagern vor allem Lunge, Leber, Nieren und Nase, bei oraler Gabe vor allem Leber und Nieren (ATSDR 2022; US EPA 2002). Mäuse sind empfindlicher als Ratten. Die Toxizität und die letale Wirkung unterscheiden sich bezüglich der untersuchten Spezies, des Geschlechts, des Stammes und des Ernährungszustands der Versuchstiere. In neueren Inhalationsstudien des NTP (2015) konnte keine NOAEC für Ratten oder Mäuse bestimmt werden. Die LOAEC betrug 25 ml/m^3^ für F344-Ratten und 6,25 ml/m^3^ für B6C3F1-Mäuse. Diese Werte sind geringer als bisher erhobene NOAEC aus älteren Studien. In oralen Langzeitstudien (104 Wochen) des NTP konnten für beide Spezies keine NOAEL abgeleitet werden. Die LOAEL für F344-Ratten und B6C3F1-Mäuse betrugen 5 bzw. 2 mg/kg KG und Tag (siehe Henschler 1979, 1986).

### 5.3 Wirkung auf Haut und Schleimhäute

#### 5.3.1 Haut

##### 5.3.1.1 In vitro

In einer Untersuchung zur Bestimmung des Permeabilitätskoeffizienten bei menschlicher Leichenhaut (Abdominalregi﻿on von mindestens drei Personen) (siehe Abschnitt 3.1) kam es nach zweistündiger okklusiver Applikation von unverdünntem, flüssigem 1,1-Dichlorethen in Kammern zu einer Hautschädigung (Verringerung des elektrischen Hautwiderstands) (Fasano und McDougal 2008).

In einer Studie aus dem Jahr 2010 mit rekonstituierter menschlicher Epidermis (RhE; EST-1000) wurde die ätzende Wirkung von 1,1-Dichlorethen (Reinheit 99 %) untersucht. Unverdünntes 1,1-Dichlorethen (50 µl) wurde topisch auf die Zellen aufgebracht, gleichmäßig verteilt und diese nach Einwirkzeiten von drei Minuten und einer Stunde untersucht (MTT-Test). Die Absorption der Lösung bei 570 nm war auf 79 bzw. 53 % im Vergleich zur Negativkontrolle herabgesetzt. Die Grenzen, die eine ätzende Wirkung anzeigen und nach drei Minuten 50 % und nach einer Stunde 15 % betragen, wurden nicht erreicht (ECHA 2023). Es liegen keine Angaben vor, ob die Testkammern abgedeckt wurden, um die Verflüchtigung des 1,1-Dichlorethens zu verhindern, daher ist das Ergebnis nur eingeschränkt für die Bewertung der Hautreizung verwendbar.

##### 5.3.1.2 In vivo

In Versuchen aus dem Jahr 1979 wirkte 1,1-Dichlorethen bei Kontakt mit der Haut von Kaninchen (k. w. A.) eine Minute nach Auftragung reizend auf die Rückenhaut; bei Auftragung auf die Ohren trat nach 20 Minuten eine Reizwirkung auf. Verschiedene Effekte wie oberflächliche Krustenbildung, nässende Ohren, Ödeme, Nekrose und Rötungen wurden berichtet (ECHA 2023). Da kommerziellen 1,1-Dichlorethenlösungen Polymerisations-Inhibitoren zugesetzt werden, vor allem *p*-Hydroxyanisol oder Phenol, ist die Hautirritation möglicherweise auf diese Antioxidantien zurückzuführen (ATSDR 2022; US EPA 1979).

#### 5.3.2 Auge

##### 5.3.2.1 In vitro

In einer Studie nach OECD-Prüfrichtlinie 437 an isolierter Rinderaugenhornhaut (BCOP-Test) wurde deren Opazität bestimmt und entweder 0,75 ml Kochsalzlösung (Negativkontrolle), 2-Ethoxyethanol (Positivkontrolle) oder 1,1-﻿Dich﻿lo﻿re﻿then aufgetropft. Nach zehn Minuten Inkubation wurden alle Hornhäute mehrfach gewaschen und die Opazität erneut bestimmt. Danach wurden alle Proben nochmals 120 Minuten inkubiert und die Opazität bestimmt. Parameter waren die Opazität und die Permeabilität, die mittels Zugabe von 1 ml Fluorescein für 90 Minuten gemessen wurde. 1,1-﻿Dich﻿lo﻿re﻿then verursachte nur eine leicht gesteigerte Opazität, aber eine statistisch signifikant erhöhte Permeabilität der Hornhaut. Als Irritationsindex wurde ein Wert von 43,9 bestimmt. Anhand einer Bewertungsskala der Reizwirkung (0–﻿3: nicht augenreizend; 3,1–25: leicht augenreizend; 25,1–55: mäßig augenreizend; 55,1–80 stark augenreizend; > 80: sehr stark augenreizend) wurde 1,1-Dichlorethen als mäßig augenreizend bewertet (ECHA 2023).

##### 5.3.2.2 In vivo

In einer Studie aus dem Jahr 1979 wurde zwei Kaninchen 50 µl unverdünntes 1,1-Dichlorethen in jeweils ein Auge getropft (k. w. A.). In das jeweils andere Auge wurde Kochsalzlösung eingebracht. Die Augen der Tiere wurden nicht gespült und nach einer Stunde, einem und acht Tagen untersucht. Es wurden zu allen Zeitpunkten Rötungen und Ödeme festgestellt (ECHA 2023). Aufgrund der unzureichenden Befundberichtung und Methodenbeschreibung ist die Studie nur eingeschränkt für eine Bewertung nutzbar.

In den in den Abschnitten 5.2.1 und 5.7.2 beschriebenen Studien mit wiederholter bis zu 105-wöchiger inhalativer Exposition gegen bis zu 100 ml/m^3^ wurde bei Sprague-Dawley-Ratten, F344-Ratten und B6C3F1-Mäusen keine augenreizende Wirkung von 1,1-Dichlorethen beobachtet (NTP 2015; Quast et al. 1986).

### 5.4 Allergene Wirkung

#### 5.4.1 Hautsensibilisierende Wirkung

##### 5.4.1.1 Tierexperimentelle Untersuchungen

Es liegt ein negatives Ergebnis aus einem Local Lymph Node Assay an CBA/Ca-Mäusen mit vier Tieren pro Gruppe vor. Mit einer 10-, 25- oder 50%igen Testzubereitung in Aceton/Olivenöl wurden Stimulationsindices von 0,84; 0,75 bzw. 0,91 bestimmt (Warbrick et al. 2001).

##### 5.4.1.2 Untersuchungen mit tierversuchsfreien Alternativmethoden (New Approach Methods (NAMs))

Zur Ableitung hautsensibilisierender Eigenschaften einer Chemikalie werden gemäß der OECD-Richtlinie 497 die Ergebnisse aus Testverfahren, die Schlüsselereignisse des Adverse Outcome Pathway (AOP) prüfen, miteinander verknüpft. Die Berücksichtigung mehrerer Testverfahren und deren Verknüpfung bei der Ableitung des sensibilisierenden Potenzials einer Chemikalie beruht auf der Annahme, dass ein Einzeltestverfahren die komplexe Abfolge bei der Entwicklung einer Sensibilisierung nicht abbilden kann.

Die experimentellen OECD-validierten Testverfahren basieren auf dem AOP für Hautsensibilisierung (OECD 2012). Methoden zur Prüfung des ersten Schlüsselereignisses („key event 1“, KE1 und „molecular initiating event“, MIE) testen die Bindungsfähigkeit an Hautproteine (elektrophile-nukleophile Interaktion). Das zweite Schlüsselereignis (KE2), eine substanzinduzierte Aktivierung von Keratinozyten, wird mit einer entsprechenden Prüfmethode analysiert. Als drittes Schlüsselereignis (KE3) wird die substanzinduzierte Reifung von dendritischen Zellen getestet. Zur Prüfung des vierten Schlüsselereignisses (KE4), die T-Zell-Proliferation, gibt es derzeit noch kein validiertes Testverfahren. Neben der experimentellen Testung der verschiedenen KEs, die auf In-chemico-Ansätzen (KE1) und In-vitro-Verfahren (KE2–﻿KE3) basieren, können auch zusätzlich Daten aus Modellen (in silico) sowie der Wirkungsmechanismus zur Bewertung herangezogen werden.

1,1-Dichlorethen wurde mit verschiedenen Methoden geprüft.

**Schlüsselereignis 1 des AOP für Sensibilisierung: Prüfung der Substanz auf Peptid-Reaktivität**

1,1-Dichlorethen wurde mit zwei OECD-validierten Methoden (Prüfrichtlinie 442C) untersucht. Im Standard Direct Peptide Reactivity Assay (**DPRA**), sowie im Amino acid Derivative Reactivity Assay (**ADRA**) wurde die Substanz negativ getestet (Natsch et al. 2013, Yamamoto et al. 2015). In einem weiteren, in Anlehnung an Prüfrichtlinie 442C durchgeführten ADRA wurde ebenfalls ein negatives Ergebnis erhalten, ebenso wie in einer Vorläufervariante des späteren DPRAs mit GSH, Lysin und Cystein (Gerberick et al. 2007). Das negative Ergebnis im Peptidreaktivitätstest steht im Einklang mit der geringen Elektrophilie von 1,1-Dichlorethen (Enoch et al. 2008).

**Schlüsselereignis 2 des AOP für Sensibilisierung: Prüfung der Substanz auf substanzinduzierte Aktivierung von Keratinozyten**

Hierzu stehen momentan verschiedene OECD-validierte Methoden (Prüfrichtlinie 442D) zur Verfügung. Im **Kerati﻿noSens** wurde 1,1-Dichlorethen negativ getestet (Natsch et al. 2013).

**Schlüsselereignis 3 des AOP für Sensibilisierung: Prüfung auf substanzinduzierte Reifung von dendritischen Zellen**

Für das dritte Schlüsselereignis stehen drei Ergebnisse zur Verfügung. Bei Testung von 1,1-Dichlorethen im Human Cell Line Activation Test (**h-CLAT**) ergaben sich ebenso wie in zwei U937 cell line activation Tests (**U-Sens,** ehemals MUSST) negative Ergebnisse (Natsch et al. 2013; OECD 2023; Piroird et al. 2015).

Zur Integration der vorliegenden Ergebnisse wurden die Integrationsansätze in Anlehnung an OECD-Richtlinie 497 beispielhaft angewandt. Im „2-von-3-Ansatz“ werden Daten aus Standard-DPRA, KeratinoSens und h-CLAT genutzt. Hierbei ergibt sich ein negatives Gesamtergebnis.

Weitere Integrationsstrategien (nach OECD-Richtlinie 497) basieren auf der Auswertung von KE1 und KE3. Zusätzlich werden in Version 1 (**ITSv1**) Daten aus computergestützten Methoden (statistische quantitative Struktur-Aktivitäts-Beziehungen, **QSAR**) und in der **ITSv2** Daten des mechanistischen Prädiktionsmodells **Derek Nexus **herangezogen.

Die Integration der Einzelergebnisse für 1,1-Dichlorethen unter Anwendung von ITSv1 oder ITSv2 ergab ebenfalls ein negatives Gesamtergebnis (OECD 2023).

#### 5.4.2 Atemwegssensibilisierende Wirkung

Es liegen keine Hinweise auf eine eigenständige atemwegssensibilisierende Wirkung vor.

Die inhalative Exposition gegen 1,1-Dichlorethen führte bereits bei nicht-sensibilisierten BALB/c-Mäusen zu einer Erhöhung von Zytokinen (IL-4, IL-5 und IL-13), wobei bei gegen Ovalbumin sensibilisierten Tieren eine Verstärkung der klassischen Symptome (Adjuvanswirkung) zu beobachten war (Ban et al. 2003, 2006).

### 5.5 Reproduktionstoxizität

Die bereits beschriebenen Daten (Henschler 1979, 1986) werden im Folgenden zusammengefasst und um neue Studien ergänzt.

#### 5.5.1 Fertilität

In einer 3-Generationen-Studie an Sprague-Dawley-Ratten mit oraler Verabreichung von 0, 50, 100 oder 200 mg 1,1-﻿Dichlorethen/l im Trinkwasser (entspricht 0, 10, 20 oder 40 mg 1,1-Dichlorethen/kg KG und Tag nach Murray et al. (1979)) über einen Zeitraum von 100 Tagen vor Verpaarung der F0-Generation (15 männliche, 30 weibliche Ratten) zeigten sich keine adversen Effekte auf die Reproduktion; als Maternaltoxizität wurden leichte Veränderungen der Leber bei den adulten Tieren bei Exposition gegen 100 oder 200 mg/l Trinkwasser festgestellt (Henschler 1979, 1986; Nitschke et al. 1983). Der NOAEL für Effekte auf die Fertilität lag bei 40 mg/kg KG und Tag, der höchsten getesteten Dosis.

In einem Dominant-Letaltest führte eine inhalative Ganzkörper-Exposition von elf männlichen CD-Ratten gegen 55 ml 1,1-Dichlorethen/m^3^ für elf Wochen (6 h/d, 5 d/Wo) zu keiner Beeinträchtigung der Fertilität und zu keinen Prä- oder Postimplantationsverlusten nach Verpaarung mit unbehandelten weiblichen Tieren (Short et al. 1977 b, siehe Abschnitt 5.6.2). In einem weiteren Dominant-Letaltest hatte die wiederholte inhalative Exposition von je 20 männlichen CD-Mäusen gegen 10 oder 30 ml/m^3^ für fünf Tage (6 h/d, Ganzkörper) keinen adversen Effekt auf die Fertilität der Tiere zur Folge. Eine fünftägige Exposition gegen 50 ml/m^3^ (6 h/d, Ganzkörper) führte zu einer statistisch signifikant geringeren Verpaarungshäufigkeit der Tiere (Anderson et al. 1977, siehe Abschnitt 5.6.2). Effekte auf die Spermatidgehalte bei F344-Ratten und der Spermienanzahl bei B6C3F1-Mäusen in Untersuchungen des NTP (2015) sind in Tabelle 1 dargestellt.

#### 5.5.2 Entwicklungstoxizität

In Studien zur pränatalen Entwicklungstoxizität nach inhalativer Exposition von Sprague-Dawley-Ratten gegen 0, 20, 80 oder 160 ml 1,1-Dichlorethen/m^3^ (7 h/d, 6.–15. Gestationstag, Ganzkörperexposition) und von Weiße-Neuseeländer-Kaninchen gegen 0, 80 oder 160 ml/m^3^ (7 h/d, 6.–18. Gestationstag, Ganzkörperexposition) zeigten sich folgende Effekte auf die embryonale und fetale Entwicklung: Nach Exposition der Muttertiere gegen 20 oder 80 ml/m^3^ entwickelten sich in Rattenfeten gewellte Rippen (Variation) und verzögerte Ossifikationen von Zentren der Halswirbel sowie des Schädels. Kaninchen, die gegen 160 ml/m^3^ exponiert waren, wiesen eine erhöhte Zahl an Resorptionen (2,7 ± 3,9; Kontrolle: 0,8 ± 1,2) auf. Alle Effekte traten nur bei gleichzeitiger Maternaltoxizität auf, die sich durch statistisch signifikant verringerte Körpergewichtszunahmen (Ratten 6.–9. Gestationstag, Kaninchen 6.–11. und 12.–18. Gestationstag) und erhöhte Lebergewichte (Ratten bei 160 ml/m^3^) zeigte (ECHA 2023; Henschler 1979; Murray et al. 1979). Die Autoren geben für die Kaninchen keine Resorptionszahlen der historischen Kontrollen an. Es ist jedoch bekannt, dass bei Kaninchen Resorptionen in einem großen Bereich auftreten können: späte Resorptionen bis 6 %, frühe Resorptionen bis 8 % (Feussner et al. 1992).

Dieselbe Arbeitsgruppe untersuchte auch die Exposition von 26 Sprague-Dawley-Ratten gegen 0 (24 Tiere) oder 200 mg 1,1-Dichlorethen/l Trinkwasser (6.–15. Gestationstag, entspricht ca. 40 mg/kg KG und Tag) vom 6. bis zum 15. Gestationstag. Der Stoff führte weder zu Maternaltoxizität noch zu adversen Effekten auf die embryonale und fetale Entwicklung (Henschler 1979, 1986; Murray et al. 1979).

Nach täglicher Exposition (die exakte Expositionsdauer pro Tag geht aus der Studie nicht hervor) von CD-Ratten gegen 0, 56 oder 283 ml 1,1-Dichlorethen/m^3^ (8.–20. Gestationstag, Ganzkörper) oder CD-Mäusen gegen 0, 15 oder 57 ml 1,1-﻿Dichlorethen/m^3^ (6.–16. Gestationstag, Ganzkörper) oder bei 23-stündiger Exposition von CD-Mäusen gegen 0, 41, 54 oder 74 ml 1,1-Dichlorethen/m^3^ (6.–15. Gestationstag, Ganzkörper) oder gegen 0, 56, 81 oder 112 ml 1,1-﻿Dich﻿lo﻿re﻿then/﻿m^3^ (6.–9., 9.–12., 12.–15., 15.–17. Gestationstag, Ganzkörper) wurden Effekte auf die Entwicklung der Feten (Skelett- und Weichteilanomalien, reduzierte Fetengewichte und Resorptionen) nur bei gleichzeitiger maternaler Toxizität festgestellt, wobei die Mäuse sensitiver gegenüber 1,1-Dichlorethen reagierten als die Ratten. Die maternale Toxizität wur﻿de anhand reduzierter Körpergewichtszunahme oder Letalität festgestellt. Nur bei Feten von CD-Mäusen, die gegen 15 ml/m^3^ exponiert waren und keine Anzeichen von Maternaltoxizität aufwiesen, wurde eine erhöhte Anzahl von nicht ossifizierten Incus (Amboss, Gehörknöchelchen) und unvollständig ossifizierten Sternebrae festgestellt. Bei den untersuchten Nachkommen der Ratten traten keine Anzeichen einer neurotoxischen Wirkung (Reflexe und gängige Verhaltenstests) von 1,1-Dichlorethen auf und die NOAEC für diese Effekte betrug 283 ml 1,1-Dichlorethen/m^3^ (Short et al. 1977 a).

In der oben beschriebenen 3-Generationen-Studie an Sprague-Dawley-Ratten traten bis zur höchsten Dosis von 40 mg 1,1-Dichlorethen/kg KG und Tag keine perinataltoxischen Effekte auf (Henschler 1986; Nitschke et al. 1983). Der NOAEL für Perinataltoxizität liegt bei 40 mg/kg KG und Tag.

Folgende Studien sind neu hinzugekommen:

Je 14 trächtige weibliche Sprague-Dawley-Ratten erhielten via direkter Infusion 1,5 oder 150 mg 1,1-Dichlorethen/l Kochsalzlösung in den Uterus während der fetalen Organentwicklung (0,5 µl Lösung/h, 7. bis 21. Gestationstag) und wurden am 22. Gestationstag (1 Tag vor Geburt) getötet und untersucht. Bei den Feten wurden Veränderungen der Herzen festgestellt (Atriumseptum, Herzkammern, Herzscheidewand). Die Inzidenz der kardialen Veränderungen betrug bei Tieren exponiert gegen 1,5 oder 150 mg 1,1-Dichlorethen/l Kochsalzlösung 12,5 oder 21 %, wohingegen die Tiere in der Kontrollgruppe eine Inzidenz von 3 % aufwiesen (Dawson et al. 1990). Da bei dieser Art der Applikation die Feten mit der Substanz direkt in Kontakt kommen können, ist diese Studie nicht zur Bewertung der entwicklungstoxischen Wirkung geeignet.

Adulten weiblichen Sprague-Dawley-Ratten wurde 1,1-Dichlorethen im Trinkwasser verabreicht (0; 0,02; 18 mg/kg KG und Tag). Den Ratten wurde 1,1-Dichlorethen entweder nur vor der Trächtigkeit verabreicht (für 82 bzw. 61 Tage) oder für 56 bzw. 48 Tage vor und zusätzlich noch für 20 Tage während der Trächtigkeit. Es wurden keine Anzeichen von Maternaltoxizität oder Effekte auf die Fetenentwicklung festgestellt. Es traten jedoch kardiale Veränderungen bei den Feten der Tiere auf, welche 1,1-Dichlorethen vor und während der Trächtigkeit erhalten hatten (Dawson et al. 1993). Nach Korrespondenz mit der US EPA wurden folgende Daten zur Studie von den Autoren nachträglich korrigiert: Die Anzahl betroffener Würfe lag in aufsteigender Dosis bei 8/11 (73 %) bzw. 13/17 (76 %), in der Kontrollgruppe bei 5/21 (24 %). Die Mittelwerte betroffener Feten pro betroffenem Wurf betrugen in aufsteigender Dosis 1,75 (entspricht 16 % der Feten im Wurf), 1,85 (entspricht 17 % der Feten im Wurf) und in der Kontrolle 1,4 (entspricht 13 % der Feten im Wurf). Die biologische Relevanz der beschriebenen Herzveränderungen wurde seitens der US EPA als fraglich bewertet. Begründet wird dies damit, dass in bisherigen Untersuchungen zur Entwicklungstoxizität von keiner anderen Forschergruppe kardiale Fehlbildungen beobachtet wurden, eine hohe Hintergrundbelastung dieser Effekte existiert, keine Dosisabhängigkeit der beobachteten kardialen Veränderungen nachweisbar ist und dass die eingesetzten Konzentrationen von Dawson et al. (1993) geringer sind als die Konzentrationen, bei denen der Metabolismus von Sprague-Daw﻿ley-Ratten bei Exposition gegen 1,1-Dichlorethen gesättigt ist. Somit wäre 1,1-Dichlorethen in der mütterlichen Leber metabolisiert worden und hätte dort mit GSH oder Makromolekülen reagiert, sodass die Ausgangsverbindung und/oder reaktive Metaboliten den Fetus wahrscheinlich nicht in nennenswerten Mengen erreicht hätten (US EPA 2002). Die Effekte auf die Herzentwicklung sind aus den aufgeführten Gründen nicht für eine Bewertung der entwicklungstoxischen Wirkung heranzuziehen.

**Fazit**: Entwicklungstoxische Effekte bei Ratten und Kaninchen treten nur bei gleichzeitiger Maternaltoxizität auf.

### 5.6 Genotoxizität

Da der Endpunkt Genotoxizität umfassend neu bewertet wird, werden bisherige Bewertungen der Kommission zum Endpunkt (Henschler 1979, 1986) neu zusammengefasst. Neue Untersuchungen sind zusätzlich angegeben.

#### 5.6.1 In vitro

##### 5.6.1.1 Bakterien

In den Salmonella-typhimurium-Stämmen TA100 und TA1530 wirkt 1,1-Dichlorethen im geschlossenen Testsystem (Luftkonzentrationen: 0,2; 2 oder 20 %) in Anwesenheit von Mikrosomen aus Leber, Nieren und Lunge der Maus oder Ratte (nicht Phenobarbital-behandelt) mutagen. Die Mutagenität war mit Mikrosomen aus der Mausleber stärker als mit Nieren- oder Lungenmikrosomen. Die mutagene Wirkung wurde mit Mikrosomen aus Leber, Nieren oder Lunge von mit Phenobarbital vorbehandelten Mäusen verstärkt (Bartsch et al. 1975). Weitere positive Resultate ergaben sich im geschlossenen System (Luftkonzentration 3 bzw. 5 %) für die Stämme TA100 und TA1535 in Anwesenheit von Lebermikrosomen aus Mäusen und Ratten, die mit Aroclor 1254 behandelt wurden. Hierbei war die mutagene Wirkung in Anwesenheit von Mikrosomen aus Nieren und Leber der Maus höher als mit Mikrosomen aus der Rattenleber (Baden et al. 1977; Jones und Hathway 1978 c). Bei Exposition gegen 2 % 1,1-Dichlorethen in der Gasphase ergaben sich positive Resultate für den Stamm TA100 mit metabolischer Aktivierung durch Lebermikrosomen aus der Maus mit und ohne Vorbehandlung durch Phenobarbital (Malaveille et al. 1977).

In Stamm TA98 wirkte 1,1-Dichlorethen in An- und Abwesenheit von Mikrosomen aus der Rattenleber mutagen (Was﻿kell 1978). In Escherichia coli K12 war 1,1-Dichlorethen nur in Anwesenheit von Mikrosomen aus der Mausleber mutagen (Greim et al. 1975). In einer vergleichenden Studie zur Bioaktivierung wirkte 1,1-Dichlorethen mutagen in den Salmonella-typhimurium-Stämmen TA92, TA98, TA100, TA1535 und TA1537 sowie in Escherichia coli WP2. Die Mutagenität war abhängig von einem Zusatz von Mikrosomen aus Leber und Nieren von C57BL/6- und Swiss-Mäusen, SD-﻿Ratten oder Chinesischen Hamstern, die zuvor inhalativ für 1–8 Tage täglich für sechs Stunden gegen 1,1-﻿Di﻿ch﻿lo﻿re﻿then exponiert waren. Es erfolgte keine Induktion mit Phenobarbital oder ähnlichen Substanzen. Die Abfol﻿ge der Stärke der mutagenen Wirkung bezüglich der Mikrosomen war: Maus (Leber, beide Stämme und Geschlechter) und Chinesischer Hamster (Leber) > Ratte (Leber) > Mensch (Leber) > Hamster (Nieren) > Maus (Nieren, beide Stämme, männlich) > Ratte (Nieren) und Maus (Nieren, weiblich). Zusätzlich waren Aktivitäten der Epoxidhydrolase und GSH-*S*-Transferase in den Nieren männlicher Swiss-Mäuse im Gegensatz zu denen weiblicher Swiss-Mäuse oder denen weiblicher und männlicher SD-Ratten nach inhalativer Exposition gegen 50 ml 1,1-Dichlorethen/m^3^ statistisch signifikant reduziert (Oesch et al. 1983). In einer neu hinzugekommenen Studie ohne metabolische Aktivierung war 1,1-Dichlore﻿then nur schwach mutagen in den Salmonella-Stämmen RSJ100 und TPT100, wohingegen die strukturell ähnliche Substanz 1,1-﻿Dichlorpropen eine etwa 5000-fach höhere Mutagenität bewirkte (Granville et al. 2005).

Vereinzelte negative oder inkonsistente Ergebnisse in Studien mit Präinkubation oder Flüssiginkubation in den Stämmen TA97, TA98, TA100, TA104, TA1535 und TA1537 mit metabolischer Aktivierung (Mortelmans et al. 1986; Stru﻿bel und Grummt 1987) sind mit der Volatilität der Substanz in nicht geschlossenen Testsystemen dieser Versuche zu erklären (ATSDR 2022). Wie bereits beschrieben, ist der Ursprung des Aktivierungssystems (Mikrosomen) bezüglich der Spezies und des Gewebes neben einer Exposition gegen die Gasphase im geschlossenen System entscheidend für eine genotoxische Aktivität von 1,1-Dichlorethen in Bakterien (Henschler 1979, 1986).

Im Saccharomyces-cerevisiae-Stamm D7 erwies sich 1,1-Dichlorethen als toxisch aber nicht mutagen ohne metabolische Aktivierung, wirkte aber mutagen und führte zu Genkonversion in Anwesenheit metabolischer Aktivierung. Zudem wurde eine Aneuploidie in den Hefestämmen D7 und D61.M mit und ohne Zusatz metabolischer Aktivierung festgestellt (Bronzetti et al. 1981; Koch et al. 1988). In Aspergillus nidulans ergab sich in einem Test auf Chromosomenfehltrennung ein positives Resultat (Crebelli et al. 1992).

##### 5.6.1.2 Säugerzellen

Die Studien zur Genotoxizität in Säugerzellen in vitro sind in Tabelle 2 aufgeführt.

In Lungenzellen des Chinesischen Hamsters (CHL) wurde nach metabolischer Aktivierung nur eine geringfügig erhöhte Anzahl von Schwesterchromatidaustauschen festgestellt (Sawada et al. 1987).

In einem Test auf DNA-Reparatursynthese (UDS) an isolierten Hepatozyten von Ratten, die mit Phenobarbital in vivo behandelt wurden, ergab sich ein positives Resultat für 1,1-Dichlorethen (Costa und Ivanetich 1984). Da die Dokumentation der Versuchsergebnisse nicht umfassend und genau erfolgte, ist diese Studie nur eingeschränkt für eine Bewertung nutzbar.

1,1-Dichlorethen führte zu einer dosisabhängigen Induktion von Chromosomenaberrationen in Hamsterlungenzellen. Es wurden hauptsächlich Chromatidbrüche und -austausche festgestellt. Eine Zugabe von GSH oder Behandlung mit Metyrapon, einem CYP-Modulator, führte zu einer starken Verminderung der Chromosomenaberrationen (Sawada et al. 1987). Dagegen ist ein negatives Resultat für Chromosomenbrüche in DON-6-Zellen des Hamsters gelistet (k. w. A., Sasaki et al. 1980 zitiert in ATSDR 2022). Daten werden nicht präsentiert und die Studie liegt der Kommission im Original nicht vor, somit kann die Inkongruenz zu den Ergebnissen von Sawada et al. (1987) nicht erklärt werden.

1,1-Dichlorethen war im Mauslymphomtest ohne metabolische Aktivierung nicht mutagen. Bei Zusatz metabolischer Aktivierung (Lebermikrosomen männlicher mit Aroclor 1254 behandelter Ratten) war 1,1-Dichlorethen ab der niedrigsten eingesetzten Konzentration von 0,16 % mutagen. Bei dieser Konzentration betrug die Zytotoxizität etwa 30 %. Die Autoren bewerteten 1,1-Dichlorethen als mutagen (McGregor et al. 1991). Die Koloniegröße wurde nicht ausgewertet.

In einem HPRT-Test und einem Na-K-ATPase-Test in V79-Zellen des Hamsters in Anwesenheit metabolischer Aktivierung (S15-Mix aus Lebern Phenobarbital-behandelter Ratten (rS15) oder Mäuse (mS15)) im geschlossenen System war 1,1-Dichlorethen nicht mutagen (Drevon und Kuroki 1979).

**Tab. 2:** Genotoxizität von 1,1-Dichlorethen in Säugerzellen in vitro

Endpunkt	Testsystem	Konzentration	wirksame Konz.^a)^	Zytotox.^a)^	Ergebnis		Literatur
					–m. A.	+m. A.	
SCE	Hamster-Lungenfibroblasten (CHL) (Exposition gegen Gasphase, 6 h, Platteninkubation für 24 h)	–m. A.: 0; 0,025; 0,05; 0,2; 1,0 mg/ml +m. A.: 0; 0,025; 0,05; 0,075; 0,1 mg/ml	+m. A.: ab 0,075 mg/ml weniger als Verdopplung, im Vergleich zur Kontrolle	keine	–	+/–	Sawada et al. 1987
DNA-Reparatursynthese (UDS)	Rattenhepatozyten (Long-Evans-Ratten, Inkubation: 2,5 h (k. w. A.)	–m. A.: 2,1 mM (k. w. A.)	k. w. A. schlecht dokumentiert; Ergebnis nicht genau nachvollziehbar	k. w. A.	+	n. u.	Costa und Ivanetich 1984
CA	Hamster-Lungenfibroblasten (CHL) (Exposition gegen Gasphase, 6 h, Platteninkubation für 18 h)	–m. A.: 0; 0,125; 0,25; 0,5; 1,0; 1,5; 2,0 mg/ml +m. A.: 0; 0,125; 0,25; 0,5; 1,0; 1,5; 2,0 mg/ml	+m. A.: 0,125 mg/ml (hauptsächlich Chromatidbrüche und -﻿austausche)	bei 2 mg/ml (+m. A.)	–	+	Sawada et al. 1987
Chromosomenbruch	Hamster (DON-6-Zellen)	k. w. A.	k. w. A.	k. w. A.	n. a.	–	Sasaki et al. 1980 zitiert in ATSDR 2022
Genmutation Tk^+/–^	Maus L5178Y-Lymphomzellen (Exposition gegen Gasphase, 4 h, Inkubation für 48 h)	–m. A.: 0, 1, 2, 4, 6, 8, 9, 12, 15, 20, 25, 30 % +m. A.: 0; 0,16; 0,31; 0,63; 1,25; 1,5; 2,0; 2,5; 3,0; 3,5 %	+m. A.: ab 0,16 %	–m. A.: keine +m. A.: ab 0,16 %	–	+	McGregor et al. 1991
Genmutation Hprt, Na^+^/K^+^-ATPase	Hamster-Lungenfibroblasten (V79) (Exposition gegen Gasphase, 5 h, Platteninkubation für 2–3 h)	–m. A.: 0, 2, 10 % +m. A.: 0, 2, 10 % S15-Mix aus Lebern von PB-beh. Ratten (rS15) u. Mäusen (mS15)	–	–m. A.: keine + rS15.: ab 2 % + mS15: keine	–	–	Drevon und Kuroki 1979

^a)^ Ergebnis statistisch signifikant, wenn nicht anders angegeben

–: negatives Ergebnis; +: positives Ergebnis; +/–: Ergebnis nicht eindeutig; CA: Chromosomenaberrationen; Hprt: Hypoxanthin-Guanin-Phosphoribosyltransferase; k. w. A.: keine weiteren Angaben; m. A.: metabolische Aktivierung; n. a.: nicht angegeben; n. u.: nicht untersucht; PB-beh.: Phenobarbital-behandelt; SCE: Schwesterchromatidaustausch; Tk: Thymidinkinase; UDS: autoradiographischer DNA-Reparatursynthesetest; Zytotox.: Zytotoxizität

#### 5.6.2 In vivo

Die Studien zur Genotoxizität in vivo sind in Tabelle 3 aufgeführt.

In zwei Host-mediated-Assays wurde der Saccharomyces-cerevisiae-Stamm D7 ins Blut (retro-orbital) männlicher CD-﻿Mäuse eingebracht, die anschließend mittels Schlundsonde entweder einmalig 400 mg 1,1-Dichlorethen/kg KG oder 22-﻿mal 100 mg 1,1-Dichlorethen/kg KG und Tag an fünf Tagen pro Woche sowie 200 mg/﻿kg KG am Tag der Untersuchung (insgesamt 23 Dosisgaben) erhielten. In den Hefen wurden sowohl Punktmutationen als auch Genkonversionen festgestellt, wenn sie aus dem Nieren- oder Lebergewebe extrahiert wurden. Für aus dem Lungengewebe extrahierte Hefezellen ergab sich ein negatives Resultat (Bronzetti et al. 1981).

In einem Comet-Assay aus dem Jahr 2016 wurde die genotoxische Wirkung in Nieren, Leber, Lunge und Knochenmark der Ratte nach dreitägiger inhalativer Exposition gegen 0, 100, 1000, 3000 oder 25 000 mg 1,1-Dichlorethen/m^3^ untersucht. Eine statistisch signifikante und biologisch relevante Induktion von DNA-Schäden bei Abwesenheit von Toxizität wurde in den Nieren bei 100, 1000 und 3000 mg/m^3^ und in der Lunge bei 1000 mg/m^3^ beobachtet. In der Leber traten erhöhte DNA-Schäden nur bei toxischen Konzentrationen (ab 1000 mg/m^3^) auf. Im Knochenmark wurde nur bei 3000 mg 1,1-Dichlorethen/m^3^ eine statistisch signifikante DNA-Schadensinduktion gemessen. Da der Effekt gering (2,2-fach) war, im Bereich der historischen Kontrollen lag und bei 25 000 mg/m^3^ kein signifikanter DNA-Schaden im Knochenmark gemessen wurde, bewerten die Autoren des REACH-Registrierungsdossiers dies als nicht biologisch relevant (ECHA 2023).

Nach sechsstündiger inhalativer Exposition gegen 40 oder 201 mg ^14^C-1,1-Dichlorethen/m^3^ wurde in den Nieren und mit geringerer Ausprägung auch in der Leber von Mäusen und Ratten eine DNA-Alkylierung mittels radioanalytischer Messung bestimmt. In den Nieren war der Effekt bei Mäusen etwa 5-fach stärker ausgeprägt als bei Ratten. Im Vergleich zu i.p. mit Dimethylnitrosamin behandelten Tieren war jedoch sowohl die DNA-Alkylierung als auch DNA-Reparatur nur sehr gering. Histologisch wurde bei Mäusen eine Nierenschädigung festgestellt. Eine gesteigerte DNA-Replikation wurde bei beiden Spezies (Ratten nur 40 mg/m^3^ untersucht) nur in den Nieren beobachtet (Reitz et al. 1980).

Nach sechs- bis neunmonatiger inhalativer Exposition gegen 1,1-Dichlorethen wurden im Knochenmark von Ratten und Mäusen keine vermehrten chromosomalen Aberrationen beobachtet (k. w. A.; Lee et al. 1977; Rampy et al. 1977). Die Ergebnisse beider Untersuchungen wurden jeweils nur ohne umfassende Versuchsdaten berichtet.

Auch in Mikronukleustests am Knochenmark von Mäusen nach einmaliger oder mehrmaliger oraler Gabe sowie im peripheren Blut nach vierzehnwöchiger inhalativer Exposition ergaben sich negative Befunde (NTP 2015; Sawada et al. 1987). Eine Toxizität im Knochenmark wurde in keiner der Studien beobachtet, obwohl bis zu hohen Dosierungen bzw. Konzentrationen getestet wurde.

Zudem wurden in einem transplazentalen Test keine erhöhten Werte an Mikronuklei in Leber und Blut von Mausfeten nach intraperitonealer Gabe an die Muttertiere induziert (Sawada et al. 1987). Studien zur Plazentagängigkeit von 1,1-﻿Dichlorethen liegen nicht vor, jedoch kann aufgrund einer nachgewiesenen Plazentagängigkeit von Trichlorethen diese auch für 1,1-Dichlorethen angenommen werden (ATSDR 2022).

Befunde zu Keimzellen in vivo sind negativ: 1,1-Dichlorethen wirkte nicht genotoxisch in einem SLRL-Versuch mit Drosophila melanogaster (Foureman et al. 1994) und in Dominant-Letaltests mit Ratte und Maus (Anderson et al. 1977; Short et al. 1977 b).

**Tab. 3:** Genotoxizität von 1,1-Dichlorethen in vivo

**Endpunkt**	**Testsystem**	**Dosis/Konzentration**	**Resultat^a)^**	**Anmerkungen^a)^**	**Literatur**
Host-mediated Assay, S. cerevisiae D7	Maus, CD, je 5 ♂ (einmalige Dosisgabe), je 4 ♂ (tägliche Gabe)	einmalig, 0, 400 mg/kg KG, Schlundsonde oder 22 d, täglich, 100 mg/kg KG, (5 d/Wo) sowie einmalig 200 mg/kg KG am Tag der Auswertung (insgesamt 23 Gaben), Schlundsonde	+ (Nieren, Leber) – (Lunge)	keine Toxizität, Ergebnisse aus gepoolten Proben des jeweiligen Organs von 3 Tieren	Bronzetti et al. 1981
**Somazellen**
DNA-Schäden, (Comet-Assay, alkalisch), Leber, Lunge, Nieren, Knochenmark	Ratte, Wistar, je 5 ♂	3 d, 4 h/d, 0, 100, 1000, 3000, 25 000 mg/m^3^, Inhalation (nur über die Nase), Untersuchung max. 4 h nach letzter Exposition	+ Leber, Lunge: ab 1000 mg/m^3^ Nieren: ab 100 mg/m^3^ – Knochenmark: nur bei 3000 mg/m^3^ geringfügig erhöht (2,2-fach) aber im Bereich der hist. Kontr.); bei 25 000 mg/m^3^ keine Schäden	Ähnlich OECD-Prüfrichtlinie 489; in Nieren u. Lunge DNA-Schäden bei nicht-toxischen Konz.; histopath. Schäden: Leber: ab 1000 mg/m^3^, Lunge: ab 3000 mg/m^3^, Nieren: ab 25 000 mg/m^3^ (keine histopath. Untersuchung des Knochenmarks)	ECHA 2023
	Maus, CD1, je 5–7 ♂ Ratte, SD, 2 ♂	6 h, 40,2 mg/m^3^ (2 Ratten, 7 Mäuse); 201,1 mg ^14^C-1,1-Dichlorethen/m^3^ (5 Mäuse), Inhalation (Ganzkörper), Positivkontrolle (Ratte): i.p., 10 mg DMN/kg KG	(+) 40,2 mg/m^3^: Maus > Ratte; Nieren > Leber; 201,1 mg/m^3^: Maus, Maximum (Nieren): 30 Alkylierungen/10^6^ Nukleotide; DMN (Ratte, Leber): 3000–4000 Alkylierungen/10^6^ Nukleotide	Sensitivität wahrscheinlich limitiert durch sehr geringe spezifische Aktivität des verwendeten ^14^C-1,1-Dichlorethen (IARC 2019); gepoolte Proben von je 2–﻿7 Tieren mehrfach analysiert	Reitz et al. 1980
DNA-Reparatur-Synthese, [^3^H]Thymidininkorporation Leber, Nieren	Maus, CD1, je 3–6 ♂	6 h, 0; 40,2; 201,1 mg/m^3^, Inhalation (Ganzkörper), Positivkontrolle (Ratte): i.p., 3, 10, 20 mg DMN/kg KG	(+) nur Nieren bei 201,1 mg/m^3^: 38 % ↑, DMN (Leber): 60–﻿637 % ↑	Toxizität: Maus (Nieren): 40,2 mg/m^3^: Dilatation, Schwellung; 201,1 mg/m^3^: Nekrose; Positivkontrolle nur für Leber angegeben	Reitz et al. 1980
DNA-Replikation, [^3^H]Thymidininkorporation, Leber, Nieren	Maus, CD1, je 3–6 ♂ Ratte, SD, je 3–6 ♂	6 h, 0; 40,2 mg/m^3^, Maus zusätzlich 201,1 mg/m^3^, Inhalation (Ganzkörper), Analyse 48 h nach Behandlungsende Positivkontrolle (Ratte): i.p., 3, 10, 20 mg DMN/kg KG	(+) Maus (Nieren): 8- bzw. 25-fach ↑ (+) Ratte (Nieren): 2,2-fach ↑ – Maus (Leber), Ratte (Leber)	Toxizität: Maus (Nieren): 40,2 mg/m^3^: Dilatation, Schwellung; 201,1 mg/m^3^: Nekrose	Reitz et al. 1980
CA, Knochenmark	Ratte, SD, je 4 ♂, ♀	6 Mo, 6 h/d, 5 d/Wo, 0, 99, 298 mg/m^3^, Inhalation (Ganzkörper)	–	nur Zusammenfassung, k. w. A.	Rampy et al. 1977
CA, Knochenmark	Ratte, SD, 54–60 ♀, 61–158 ♂, Maus, CD1, je 30 ♂, ♀	Ratte: 9 Mo, 6 h/d, 5 d/Wo, 0, 221 mg/m^3^, Inhalation (Ganzkörper), Maus: 6 Mo 6 h/d, 5 d/Wo, 0, 221 mg/m^3^, Inhalation (Ganzkörper)	–		Lee et al. 1977
MN, Knochenmark	Maus, ddY, je 6 ♂	einmalig, 0, 25, 50, 100, 200 mg/kg KG oder 4 d, 0, 25, 50, 100 mg/kg KG u. d, in Olivenöl, Schlundsonde	–	bei einmaliger Dosis von 200 mg/﻿kg KG starben 3 von 6 Tieren, bei viermaliger täglicher Gabe von 100 mg/kg starb 1 von 6 Tieren, 1000 PCE/Tier ausgewertet, keine Tox. beobachtet [PCE (%)]	Sawada et al. 1987
MN, fötale Leber und Blut	Maus, ICR, je 4 für 0 bzw. 25 mg/﻿kg KG, je 6 für 50 bzw. 100 mg/kg KG	einmalig, 0, 25, 50, 100 mg/kg KG, i.p.-Gabe an die Muttertiere am 18. GD, Analyse 24 h nach Gabe	–	keine Tox. beobachtet [PCE (%)]	Sawada et al. 1987
MN, peripheres Blut	Maus, B6C3F1/N, je 5 ♀, ♂	14 Wo, 6 h/d, 5 d/Wo, 0; 25,1; 50,3; 100,6; 200,1 mg/m^3^; ♀ auch 402,2 mg/m^3^, Inhalation (Ganzkörper)	–	keine Tox. beobachtet [PCE (%)]	NTP 2015
Genmutation *Trp53* Codon 123–303, Nierentumoren	Maus, B6C3F1/N, je 3 ♂	2 a, 6 h/d, 5 d/Wo, 0; 50,2; 201,1 mg/m^3^ Inhalation (Ganzkörper)	–		NTP 2015
**Keimzellen**
Genmutation (SLRL), Keimzellen	D. melanogaster ♂	72 h, 0, 20 000, 25 000 mg/kg, Futter, 5000 mg/kg, Injektion (k. w. A.)	–	k. A. zur Tox.; > 3000 X-﻿Chromosomen untersucht, Mortalität 17 % nach Gabe von 25 000 mg/﻿kg Futter	Foureman et al. 1994
Dominant-Letaltest	Maus, CD-1, 50 ♂ (Kontrolle), je 20 ♂	1 Wo, 6 h/d, 5 d/Wo, 0; 40,2; 120,6; 201,1 mg/m^3^; Inhalation (Ganzkörper), verpaart 8 Wo mit 2 ♀, jeweils 5 d, Untersuchung der ♀ an GD 13	–	Dosiswahl nach vorangegangenen Toxizitätsstudien	Anderson et al. 1977
Dominant-Letaltest	Ratte, CD, 11 ♂	11 Wo, 6 h/d, 5 d/Wo, 0; 221,2 mg/m^3^, Inhalation (Ganzkörper), 1 Wo verpaart mit 2 ♀, Untersuchung der ♀ an GD 13	–	Dosiswahl wie bei Lee et al. 1977	Short et al. 1977 b

^a)^ Ergebnis statistisch signifikant, wenn nicht anders angegeben

–: negatives Ergebnis; +: positives Ergebnis; (+): schwach positives Ergebnis; a: Jahr; CA: Chromosomenaberrationen; d: Tag; DMN: Dimethylnitrosa﻿min; GD: Gestationstag; h: Stunde; hist. Kontr.: historische Kontrollen; histopath.: histopathologisch; i.p.: intraperitoneal; k. A.: keine Angaben; Konz.: Konzentrationen; k. w. A.: keine weiteren Angaben; max.: maximal; MN: Mikronuklei; Mo: Monat; SLRL: Sex-linked recessive lethal test; Tox.: Toxizität; Wo: Woche

##### Untersuchungen von Tumorgewebe der 2-Jahre-Inhalationsstudie des NTP

In Nierenzellkarzinomen von je drei männlichen B6C3F1-Mäusen der 12,5- und 25-ml/m^3^-Konzentrationsgruppe aus der NTP-Kanzerogenitätsstudie (NTP 2015; siehe Abschnitt 5.7.2) wurde eine erhöhte Genexpression von *Trp53* mittels Microarray und PCR und ebenfalls eine nukleäre Induktion von P53-Protein festgestellt (siehe auch Abschnitt 2). Eine Analyse der Hotspot Codon-Regionen (Codons 123–303) in *Trp53* ergab jedoch keine erhöhten Mutationshäufigkeiten (Hayes et al. 2016). Immunhistochemisch wurde multifokale nukleäre Expression von γH2AX in den Nierenzellkarzinomen gezeigt, was ein Hinweis auf DNA-Doppelstrangbrüche ist (Hayes et al. 2016). Da es sich hier um Tumorgewebe handelt, kann das Vorhandensein von DNA-Doppelstrangbrüchen nicht als Hinweis auf einen genotoxischen Wirkungsmechanismus zur Kanzerogenität gewertet werden.

Riva et al. (2020) untersuchten Tumoren von B6C3F1-Mäusen aus NTP-Studien mit 20 Substanzen, welche bei der IARC als (potentiell) krebserregend für den Menschen (Kategorie 1, 2A oder 2B) klassifiziert sind, auf somatische Mutationen mittels Sequenzierung. Für 1,1-Dichlorethen wurden fünf bronchiolo-alveoläre Karzinome (insgesamt drei männliche Tiere; eins der 12,5- und zwei der 25-ml/m^3^-Gruppe sowie zwei weibliche Tiere (Gruppenzugehörigkeit nicht genau nachvollziehbar, mindestens ein Tier der 12,5-ml/m^3^-Gruppe)), sieben hepatozelluläre Karzinome (fünf männliche und zwei weibliche Tiere der 25-ml/m^3^-Gruppe) und sechs Nierenzellkarzinome (sechs männliche Tiere; eins der 12,5-ml/m^3^-Gruppe, fünf der 25-ml/m^3^-Gruppe) sequenziert. Als Kontrollen wurden entsprechende spontan aufgetretene Leber- und Lungentumoren von unbehandelten Tieren sowie tumorfreie Gewebe von 29 Mäusen verwendet (es wurde auch „gematchtes“ tumorfreies Gewebe von zwei Mäusen mit 1,1-Dichlorethen-induzierten Lungentumoren verwendet). Nur die spontan aufgetretenen Lebertumoren stammten von Kontrolltieren derselben NTP-Studie, die spontanen Lungentumoren aus anderen NTP-Studien. Kontrolltumoren der Nieren lagen nicht vor.

Die Häufigkeit an somatischen Einzelnukleotid-Varianten („single nucleotide variants“) war in den durch 1,1-Dichlorethen induzierten Tumoren von Leber und Lunge nicht signifikant unterschiedlich zur Kontrolle. In Nierentumoren war die Anzahl an Mutationen jedoch etwa sechsfach höher. Da Kontrolltumoren für die Nieren nicht vorlagen, konnte keine Signifikanz berechnet werden. Insgesamt war für 17 von 20 getesteten Substanzen die somatische Mutationsrate in Substanz-induzierten und spontanen Tumoren von unbehandelten Kontrolltieren nicht signifikant unterschiedlich.

Mittels hierarchischer Clusteranalyse wurden aus den Mutationsspektren Mutationssignaturen extrahiert und mit den Signaturen für Einzelbasensubstitutionen (SBS = Single Base Substitution) in humanen Tumoren aus der COSMIC (Catalogue Of Somatic Mutations In Cancer)-Datenbank (Alexandrov et al. 2020; Sanger Institute 2023) verglichen. Für 1,1-﻿Dichlorethen wurden zwar verschiedene SBS extrahiert, jedoch war eine davon (mSBS_N1) sehr spezifisch für diese Substanz und wurde fast ausschließlich in 1,1-Dichlorethen-induzierten Tumoren mit steigendem Anteil an den Gesamtmutationen in Lunge, Leber und Nieren beobachtet. In den Nierentumoren gehen die meisten Mutationen auf Signatur mSBS_N1 zurück. In Leber- und Lungentumoren traten verschiedene mutagene Prozesse (auch mSBS_N1) etwa gleichwertig häufig auf. Nur in 1,2,3-Trichlorpropan-induzierten-Tumoren trat diese Signatur ebenfalls auf, verursachte dort jedoch nur einen minimalen Anteil der Mutationen (< 5 % der Mutationen). Die Signatur ist durch T > A- und T > C-Substitutionen gekennzeichnet und zeigt in den Nieren, jedoch nicht in Leber und Lunge, einen „transcriptional strand bias“ mit mehr Mutationen von A als T auf dem nicht-transkribierten DNA-Strang von Genen, was auf eine Schädigung von Adeninbasen und einer anschließenden DNA-Reparatur durch transkriptionsgekoppelte Nukleotidexzisionsreparatur hindeutet. In COSMIC ist noch keine äquivalente Signatur in humanen Tumoren beschrieben (Sanger Institute 2023).

Die weiteren extrahierten Signaturen mSBS1, mSBS5, mSBS12, mSBS17, mSBS18, mSBS19 und mSBS40 waren nicht spezifisch und wurden sowohl in Kontrolltumoren als auch in anderen Chemikalien-induzierten Tumoren beobachtet. Dass sie auch beim Menschen sowohl in Tumorgewebe als auch in nicht transformiertem Gewebe beobachtet wurden (Alexandrov et al. 2015), deutet auf einen endogenen Prozess hin. Die Ätiologie der Mutationssignaturen mSBS5, mSB12, mSB17, mSB19 und mSB40 ist unbekannt. Signatur 18 wird auf reaktive Sauerstoffspezies zurückgeführt und Signatur 1 entsteht über enzymatische Desaminierung von 5-Methylcytosin zu Thymin. In einem 1,1-Dichlorethen-induzierten Lebertumor wurde zusätzlich noch eine neue beim Menschen unbekannte Mutationssignatur (mSBS_N3) entdeckt, welche jedoch nicht spezifisch war, sondern auch durch andere Substanzen (Cobalt, Antiepileptikum Primidon, Flammenschutzmittel DE-71 Pentabromdiphenylether-Mischung) induziert oder in einem Spontan-Tumor identifiziert wurde. Die Signatur hatte Ähnlichkeit mit durch APOBEC-induzierten Mutationen („Apolipoprotein B mRNA Editing Enzyme, Catalytic Polypeptide-like“).

Außer bei Signatur mSBS_N1 war bei Kontrollen und bei 1,1-Dichlorethen-exponierten Tieren der relative Beitrag der Signaturen ähnlich. Mit hierarchischer Clusteranalyse wurde eine hohe Ähnlichkeit (clustering) zwischen 13 von 18 1,1-﻿Dichlorethen-induzierten Tumoren (drei Lungentumoren und zwei Lebertumoren fielen aus diesem Cluster) nachgewiesen. Auch die Häufigkeit an Dinukleotid-Substitutionsmutationen und INDEL (Insertionen und Deletionen)-Mutationshäufigkeiten waren in den 1,1-Dichlorethen-induzierten Nierentumoren ausgeprägt (aufgrund fehlender Kontrollen ist kein genauer Vergleich möglich). Leber und Lunge waren bezogen auf die Dinukleotid-Mutationen oder INDELs nicht signifikant verschieden von Kontrolltumoren. Auch diese Mutationssignaturen waren nicht unterschiedlich zu anderen Tumoren. Ebenfalls wurden die Orthologen von 299 humanen „hotspot driver Genmutationen“ für Krebs analysiert. Durch 1,1-Dichlorethen-induzierte Lungentumoren wiesen Missense-Mutationen in *Kras* (Q61R) und *Fgfr2* (C401R) auf, wogegen in den Lebertumoren verschiedene Missense-Mutationen in *Hras* zu finden waren. Dieselbe Art an Mutationen trat auch in Kontrolltumoren auf. Nierentumoren hatten dagegen Driver-Mutationen in *Keap1*, *Ddr2* und *Tnfaip3*. Nach Berechnungen waren verschiedene Driver auf die Signatur mSBS_N1 zurückzuführen. Dasselbe Muster an Deletionen in den Chromosomen 4, 12 und 13 trat in allen Nierentumoren auf. Als Besonderheit merkten die Autoren weiterhin an, dass ein Lungentumor in der 1,1-Dichlorethen-Gruppe Chromothripsis (eine Vielzahl von Umlagerungen von Chromosomenabschnitten) zeigte, was sonst nur für einen weiteren Chemikalien-induzierten Tumor und in keinem Spontan-Tumor beobachtet wurde. Untersuchung der Daten von ca. 24 000 humanen Tumorgenomen auf Präsenz der Signatur mSBS_N1 zeigte eine Assoziation mit Plattenepithelkarzinomen der Lunge und Harnblasenübergangsepithelkarzinomen.

Zusammenfassend wiesen Nierentumoren sehr viel höhere Mutationshäufigkeiten auf als Lungen- und Lebertumoren und auch der Anteil an Mutationssignaturen (und damit der Prozess der Tumorigenese) war gewebespezifisch. Jedoch wurde eine 1,1-Dichlorethen-spezifische Mutationssignatur in allen drei untersuchten Tumorgeweben beobachtet sowie das Auftreten von verschiedenen Driver-Mutanten auf diesen mutagenen Prozess zurückgeführt (Riva et al. 2020). Einschränkend ist anzuführen, dass keine Kontrolltumoren für die Nieren vorlagen und die Kontroll-Lungentumoren aus anderen NTP-Studien stammten. Jedoch zeigten die aus verschiedenen NTP-Studien stammenden Kontrolltumoren gro﻿ße Übereinstimmung bezüglich der Mutationssignaturen untereinander.

#### 5.6.3 Zusammenfassung

1,1-Dichlorethen wirkte in vitro überwiegend nur nach metabolischer Aktivierung klastogen in Säugerzellen sowie mutagen in Hefen und Bakterien. Mikrosomen aus der Leber oder den Nieren von Mäusen führten zu stärkerer Aktivierung als Mikrosomen aus der Ratte oder dem Menschen (Leber). Gleichzeitig reduzierte 1,1-Dichlorethen Entgiftungsmechanismen (Epoxidhydrolase und GSH-*S*-Transferase) in den Nieren der männlichen Maus. Dies unterstreicht die enzym- und gewebespezifische Aktivierung von 1,1-Dichlorethen im Metabolismus zu reaktiven Intermediaten und deren Zusammenhang mit der adversen Wirkung (siehe Abschnitt 2 und 3). Ein Mauslymphomtest war positiv, je ein Hprt- und Na-K-ATPase-Test negativ. Erklärungen hierfür könnten ihre geringere Sensitivität oder der Einsatz verschiedener Aktivierungssysteme (Phenobarbital- oder Aroclor-induziert) sein. In vivo zeigte sich die mutagene Wirkung auf Hefen auch im Host-mediated Assay an der Maus.

DNA-Alkylierung und DNA-Reparatursynthese in Nieren und Leber männlicher Mäuse und Ratten waren minimal, in den Nieren von Mäusen am stärksten, wobei jedoch gleichzeitig Gewebeschäden und eine deutlich gesteigerte DNA-Replikation auftraten. Bei fehlender Toxizität kam es in einem Comet-Assay mit Ratten nach Inhalation zu DNA-Schäden in der Lunge bei 10-fach höheren Konzentrationen als in den Nieren. Im Knochenmark wurden nach oraler oder inhalativer Exposition bei Ratten und Mäusen keine Chromosomenaberrationen, Mikronuklei oder DNA-Schäden (Comet) und, falls untersucht, auch keine Toxizität beobachtet. Bei transplazentaler Gabe wurden ebenfalls keine Mikronuklei in Feten festgestellt. Eine Mutationsanalyse mittels Genomsequenzierung von Lungen-, Leber- und Nierentumoren der Mäuse aus der NTP-Kanzerogenitätsstudie zeigte vermehrt Mutationen in den Nieren und eine 1,1-﻿Dichlore﻿then-spezifische Mutationssignatur in allen Geweben mit stärkster Ausprägung, ebenfalls in den Nieren. Valide Studien an Keimzellen und Dominant-Letaltests an Maus und Ratte verliefen negativ.

### 5.7 Kanzerogenität

#### 5.7.1 Kurzzeitstudien

In einem Initiations-Promotions-Versuch wirkte 1,1-Dichlorethen als Initiator an der Haut, war aber kein vollständiges Kanzerogen bei dermaler oder subkutaner Gabe an Mäuse. Jedoch ist nicht ersichtlich, ob der Vergleich gegen die Lösungsmittelkontrollen oder gegen die Promotor-Kontrollen durchgeführt wurde. Außerdem rief der Promotor selbst schon Papillome an der Haut hervor, so dass die Initiator-Wirkung von 1,1-Dichlorethen nicht gesichert ist (Henschler 1979, 1986). Neue Studien liegen nicht vor.

#### 5.7.2 Langzeitstudien

Studien nach chronischer Exposition, in denen Tumorbefunde beschrieben werden, sind in der Begründung und einem Nachtrag ausführlich dargestellt (Henschler 1979, 1986).

Die negativen Studien an der Ratte und der Maus sind für die Bewertung der kanzerogenen Wirkung nur eingeschränkt verwertbar, da sie heutigen Standards für Kanzerogenitätsstudien nicht entsprechen: z. T. ist die Tierzahl sehr klein, die Reinheit der Verbindung ist nicht angegeben oder eine statistische Auswertung fehlt.

In einer 2-Jahre-Schlundsonden-Studie an Ratten (0, 1, 5 mg/kg KG und Tag) und Mäusen (0, 2, 10 mg/kg KG und Tag) traten bei den männlichen Ratten in der höchsten Dosisgruppe statistisch signifikant mit einem positiven Trend erhöhte Inzidenzen an Phäochromozytomen und Hodentumoren auf, sowie subkutane Fibrome und Inselzelltumoren im Pankreas (nur Trend-Test statistisch signifikant) und bei den weiblichen Ratten ein statistisch signifikanter dosisabhängiger Anstieg der Hypophysenadenome. Bei den männlichen Mäusen wurden keine erhöhten Tumorinzidenzen beobachtet und bei den weiblichen waren Lymphome und Leukämien statistisch signifikant in der mittleren Dosisgruppe erhöht. Von den Autoren wird darauf hingewiesen, dass die maximal tolerierbare Dosis wahrscheinlich nicht erreicht worden ist. Es war keine eindeutig kanzerogene Wirkung für die Ratte und die Maus ableitbar (NTP 1982).

Seit der Bewertung in der letzten Begründung und dem Nachtrag (Henschler 1979, 1986) sind neue Studien hinzugekommen:

Je 84–86 männliche und weibliche SD-Ratten (6–7 Wochen alt) wurden gegen 0, 10 oder 40 ml 1,1-Dichlorethen/m^3^ (99 % Reinheit, stabilisiert mit *p*-Hydroxyanisol) für sechs Stunden pro Tag an fünf Tagen in der Woche exponiert. In einer Zwischenuntersuchung nach vier Wochen an zusätzlichen Tieren traten keine Befunde auf. Daraufhin wurden die Konzentrationen auf 25 bzw. 75 ml/m^3^ erhöht und die Tiere 18 Monate exponiert und sechs Monate nachbeobachtet. In beiden exponierten Tiergruppen wurde eine Lungenentzündung, bedingt durch eine Infektion mit Mycoplasma pulmonis, festgestellt und in deren Folge war die Mortalität ab dem 15. Monat bei den exponierten Tieren signifikant höher als in der Kontrollgruppe. Es wurden keine statistisch signifikant erhöhten Inzidenzen von Neoplasien festgestellt (Quast et al. 1986). Aufgrund der hohen Mortalität ist die Studie nur eingeschränkt zur Bewertung geeignet.

Je 60 bzw. 54 weibliche SD-Ratten (13 Wochen alt) wurden gegen 0 oder 100 ml 1,1-Dichlorethen/m^3^ inhalativ lebenslang Ganzkörper-exponiert. Die Reinheit der Testsubstanz betrug mehr als 99 %. Die Exposition erfolgte an vier Stunden pro Tag an fünf Tagen in der Woche für einen Zeitraum von sieben Wochen und anschließend für sieben Stunden pro Tag an fünf Tagen in der Woche für einen Zeitraum von 97 Wochen. Es wurden Tumoren der Brustdrüsen (benigne und maligne), Leukämien, Phäochromozytome und Phäochromoblastome festgestellt. Gesondert wurden nur die Tumoren der Brustdrüse aufgeführt: Maligne Tumoren bei den Kontrolltieren bzw. exponierten Tieren traten in folgender Inzidenz auf: Kontrolle: 2/60 Tieren (entspricht 3,3 %); exponierte Tiere: 4/54 Tieren (entspricht 7,4 %) (Cotti et al. 1988). Aufgrund der unzureichenden Befundberichtung (Überlebenszahlen fehlen, keine genaue Beschreibung der Tumoren, keine statistische Bewertung der Befunde) und nur einer getesteten Konzentration ist die Studie nur eingeschränkt für eine Bewertung geeignet.

In einer NTP-Studie wurden F344-Ratten (jeweils 5–6 Wochen alt) 105 Wochen lang gegen 0, 25, 50 oder 100 ml/m^3^ und B6C3F1-Mäuse gegen 0; 6,25; 12,5 oder 25 ml/m^3^ Ganzkörper-exponiert (Reinheit 99,9 %). Bei den weiblichen Ratten und den Mäusen war die Mortalität in der höchsten Konzentrationsgruppe statistisch signifikant erhöht. Nichtneoplastische Effekte sind in Abschnitt 5.2.1 beschrieben. Die einzelnen Befunde zur Kanzerogenität sind in Tabelle 4 und 5 dargestellt.

Bei Ratten traten in der Nase Adenome des respiratorischen Epithels auf, deren Inzidenz zwar knapp nicht statistisch signifikant erhöht war, die aber als substanzbedingt angesehen werden. Bei weiblichen Ratten waren die Inzidenzen für C-Zelladenome und -karzinome der Schilddrüse ab der niedrigsten Konzentration und die der mononukleären Zellleukämie in der höchsten Konzentrationsgruppe statistisch signifikant erhöht. Die Inzidenzen für renale Tubuluskarzinome männlicher Ratten waren nicht statistisch signifikant erhöht, aber aufgrund ihrer Seltenheit substanzbedingt. Maligne Mesotheliome ausgehend von der Tunica vaginalis traten in allen Expositionsgruppen der männlichen Ratten vermehrt auf (NTP 2015).

Bei den männlichen Mäusen waren die Inzidenzen von Nierenadenomen und -karzinomen ab der niedrigsten Konzentration statistisch signifikant erhöht. Weibliche Mäuse wiesen statistisch signifikant erhöhte Inzidenzen von hepatozellulären Karzinomen sowie von bronchiolo-alveolären Karzinomen auf. Die Inzidenz der seltenen Hepatocholangiokarzinome war erhöht, jedoch nicht statistisch signifikant. Nach Exposition gegen die höchste Konzentration traten statistisch signifikant erhöhte Inzidenzen von Hämangiosarkomen der Leber sowie Hämangiome und Hämangiosarkome (kombiniert) in verschiedenen Organen weiblicher Mäuse auf (NTP 2015).

**Tab. 4:** Kanzerogenitätsstudie mit 1,1-Dichlorethen an Ratten (NTP 2015)

Autor:	NTP 2015				
Stoff:	1,1-Dichlorethen (Reinheit 99,9 %)				
Spezies:	**Ratte**, F344/N, 50 ♂, 50 ♀ pro Gruppe				
Applikation:	Inhalation				
Konzentration:	0, 25, 50, 100 ml/m^3^				
Dauer:	105 Wochen, 5 d/Wo, 6 h/d				
Toxizität:	ab 25 ml/m^3^: Nasenturbinalien: ♂/♀: chron. Entzündung, Atrophie u. Hyperostose, ♀: Hyperplasie des resp. Epithels; Lunge: ♂: Hyperplasien im alv. Epithel; Leber: ♂/﻿♀: chron. Entzündung, diffuse fettige Veränderungen; Ovarien: ♀: Dilatation d. Bursa ovarica; Mesenterium: ♀: Fettgewebsnekrosen; Bauchfell: (siehe Abschnitt 5.2.1);				
		**Konzentration [ml/m^3^]**			
		**0**	**25**	**50**	**100**
Überlebende	♂	25/50 (50 %)	27/50 (54 %)	22/50 (44 %)	19/50 (38 %)
	♀	30/50 (60 %)	26/50 (52 %)	30/50 (60 %)	19/50 (38 %)*
**Tumoren und Präneoplasien**					
**maligne Mesotheliome:**					
ausgehend von Tunica vaginalis: alle Organe betroffen (Darm, Mesenterium, Bauchspeicheldrüse, Prostata, Milz und Leber)	♂	1/50 (2 %)	12/50 (24 %)***	28/50 (56 %)***	23/50 (46 %)***
	♀	0/50 (0 %)	1/50 (2 %)	1/50 (2 %)	0/50 (0 %)
**Schilddrüse (C-Zellen):**					
Adenome	♀	3/50 (6 %)	4/50 (8 %)	6/48 (13 %)	11/50 (22 %)*
Karzinome	♀	0/50 (0 %)	6/50 (12 %)*	2/48 (4 %)	2/50 (4 %)
Adenome/Karzinome (kombiniert)^a)^	♀	3/50 (6 %)	10/50 (20 %)*	8/48 (17 %)	13/50 (26 %)**
**Blut:**					
mononukleäre Zellleukämie	♀	10/50 (20 %)	11/50 (22 %)	13/50 (26 %)	25/50 (50 %)***
**Nieren^b)^:**					
Tubulushyperplasien	♂	3/50 (6 %)	5/50 (10 %)	6/49 (12 %)	8/50 (16 %)
	♀	1/50 (2 %)	2/50 (4 %)	0/50 (0 %)	2/50 (4 %)
Tubulusadenome	♂	3/50 (6 %)	3/50 (6 %)	5/49 (10 %)	1/50 (2 %)
	♀	0/50 (0 %)	0/50 (0 %)	1/50 (2 %)	0/50 (0 %)
Tubuluskarzinome	♂	0/50 (0 %)	2/50 (4 %)	1/49 (2 %)	1/50 (2 %)
Tubulusadenome/-karzinome (kombiniert)	♂	3/50 (6 %)	4/50 (8 %)	6/49 (12 %)	2/50 (4 %)
**Nase:**					
Hyperplasien (resp. Epithel)	♂	5/50 (10 %)	8/50 (16 %)	22/50 (44 %)**	31/50 (62 %)**
	♀	4/50 (8 %)	12/50 (24 %)*	14/50 (28 %)**	27/50 (54 %)**
Metaplasien (olf. Epithel, resp.)	♂	3/50 (6 %)	49/50 (98 %)**	49/50 (98 %)**	48/50 (96 %)**
	♀	1/50 (2 %)	50/50 (100 %)**	50/50 (100 %)**	50/50 (100 %)**
Entzündung (chronisch, olf. u. resp. Epithel)	♂	9/49 (18 %)	36/50 (72 %)**	45/50 (90 %)**	48/50 (96 %)**
	♀	7/50 (14 %)	45/50 (90 %)**	46/50 (92 %)**	46/50 (92 %)**
Adenome (resp. Epithel)^c)^	♂	0/49 (0 %)	0/50 (0 %)	1/50 (2 %)	4/50 (8 %)^d)^
	♀	0/50 (0 %)	0/50 (0 %)	0/50 (0 %)	1/50 (2 %)
**Lunge:**					
Hyperplasien (alv. Epithel)	♂	7/49 (14 %)	18/50 (36 %)*	14/50 (28 %)	14/50 (28 %)

*p < 0,05;

**p < 0,01;

***p < 0,001

alv.: alveolär; olf.: olfaktorisch; resp.: respiratorisch

^a)^ Inzidenz in historischen Kontrollen aus Inhalationsstudien: 14/200 (7,0 ± 1,2 %), Bereich 6–8 %; alle Expositionspfade: 87/690 (12,7 ± 5,8 %), Bereich 6–22 % (NTP 2015)

^b)^ Einzel- und Stufenschnitte (kombiniert)

^c)^ ♂: Inzidenz in historischen Kontrollen aus Inhalationsstudien: 0/198; alle Expositionspfade: 0/697; ♀: 0/200; alle Expositionspfade: 1/697 (0,1 ± 0,5 %), Bereich 0–2 % (NTP 2015)

^d)^ p = 0,051

**Tab. 5:** Kanzerogenitätsstudie mit 1,1-Dichlorethen an Mäusen (NTP 2015)

Autor:	NTP 2015					
Stoff:	1,1-Dichlorethen (Reinheit 99,9 %)					
Spezies:	**Maus**, B6C3F1, 50 ♂, 50 ♀ pro Gruppe					
Applikation:	Inhalation					
Konzentration:	0; 6,25; 12,5; 25 ml/m^3^					
Dauer:	105 Wochen, 5 d/Wo, 6 h/d					
Toxizität:	ab 6,25 ml/m^3^: ♀: abnormale Atmung, vermindertes Körpergewicht, Abmagerung; Nasenturbinalien: ♂/♀: Atrophie, Hyperostosen; olf. Epithel: ♂/﻿♀: Metaplasien; Nieren: ♂: Tubulushyperplasien, Zysten; Lunge: ♂: Hyperplasien im alv. Epithel; Mesenterium: ♀: Fettgewebsnekrosen (siehe Abschnitt 5.2.1)					
		**Konzentration [ml/m^3^]**			
		**0**	**6,25**	**12,5**	**25**
Überlebende	♂	29/50 (58 %)	40/50 (80 %)	32/50 (64 %)	19/50 (38 %)*
	♀	36/50 (72 %)	25/50 (50 %)*	30/50 (60 %)	24/50 (48 %)*
**Tumoren und Präneoplasien**					
**Nieren:**					
Nephropathie	♂	44/50 (88 %)	46/50 (92 %)	37/50 (74 %)	44/50 (88 %)
Tubulushyperplasien	♂	0/50 (0 %)	8/50 (16 %)***	22/50 (44 %)***	16/50 (32 %)***
Tubulusadenome	♂	0/50 (0 %)	5/50 (10 %)*	19/50 (38 %)***	10/50 (20 %)***
Tubuluskarzinome^a)^	♂	0/50 (0 %)	7/50 (14 %)*	31/50 (62 %)***	18/50 (36 %)***
Tubulusadenome/-karzinome (kombiniert)	♂	0/50 (0 %)	11/50 (22 %)***	37/50 (74 %)***	27/50 (54 %)***
**Leber:**					
Adenome	♂	37/50 (74 %)	35/50 (70 %)	33/50 (66 %)	25/50 (50 %)
	♀	25/50 (50 %)	21/50 (42 %)	36/50 (72 %)**	29/50 (58 %)
Karzinome	♂	26/50 (52 %)	19/50 (38 %)	15/50 (30 %)	29/50 (58 %)
	♀	8/50 (16 %)	14/50 (28 %)	12/50 (24 %)	17/50 (34 %)*
Adenome/Karzinome (kombiniert)	♂	44/50 (88%)	41/50 (82%)	41/50 (82%)	42/50 (84%)
	♀	28/50 (56 %)	30/50 (60 %)	37/50 (74 %)*	38/50 (76 %)*
Hepatocholangiokarzinome	♂	1/50 (2 %)	2/50 (4 %)	2/50 (4 %)	3/50 (6 %)*
	♀	0/50 (0 %)	1/50 (2 %)	1/50 (2 %)	2/50 (4 %)
**Hämangiome und Hämangiosarkome:**					
Leber	♀	1/50 (2 %)	1/50 (2 %)	1/50 (2 %)	6/50 (12 %)*
alle Organe^b)^	♀	4/50 (8 %)	6/50 (12 %)	6/50 (12 %)	11/50 (22 %)*
**Lunge:**					
Hyperplasien	♂	3/50 (6 %)	7/50 (14 %)	4/50 (8 %)	6/50 (12 %)
Karzinome (bronchiolo-alv.)	♀	1/50 (2 %)	2/50 (4 %)	7/50 (14 %)*	5/49 (10 %)
**Dünndarm:**					
Adenome und Karzinome^c)^	♂	1/50 (2 %)	3/50 (6 %)	1/50 (2 %)	2/50 (4 %)
	♀	2/50 (4 %)	1/50 (2 %)	2/50 (4 %)	4/50 (8 %)

*p < 0,05;

**p < 0,01;

***p < 0,001

alv.: alveolär; olf.: olfaktorisch

^a)^ Inzidenz in historischen Kontrollen aus Inhalationsstudien: 0/298; alle Expositionspfade: 3/944 (0,3 ± 1,0 %), Bereich 0–4 % (NTP 2015)

^b)^ Inzidenz in historischen Kontrollen aus Inhalationsstudien: 21/300 (7,0 ± 2,1 %), Bereich 4–10 %; alle Expositionspfade: 55/950 (5,8 ± 3,4 %), Bereich 2–14 % (NTP 2015)

^c)^ ♂: Inzidenz in historischen Kontrollen aus Inhalationsstudien: 10/300 (3,3 ± 2,7 %), Bereich 0–8 %; alle Expositionspfade: 31/950 (3,3 ± 2,3 %), Bereich 0–8 %; ♀: 4/300 (1,3 ± 1,6 %), Bereich 0–4 %; alle Expositionspfade: 10/950 (1,1 ± 1,4 %), Bereich 0–4 % (NTP 2015)

#### 5.7.3 Humanrelevanz der Befunde

Die innerhalb der Studie des NTP vorgenommenen Untersuchungen weisen auf statistisch signifikante Unterschiede der Genexpression in Mesotheliomen von gegen 1,1-Dichlorethen exponierten Tieren im Vergleich zu Mesotheliomen in der Kontrollgruppe sowie denen in historischen Kontrollen hin (NTP 2015). Diese Ergebnisse werden in Abschnitt 2 diskutiert. Mesotheliome, ausgehend von der Tunica vaginalis, werden, ebenso wie die mononukleäre Zellleukämie in F344-Ratten, als nicht humanrelevant angesehen (Laube et al. 2019).

### 5.8 Sonstige Wirkungen

Ein statistisch signifikanter Anstieg der Produktion von T-Helferzellen des Typs 2 (Th2) (IL-4, IL-5, IL-13 und Interferon-γ) in Einzelzellsuspensionen, die am 25. Tag aus lungenassoziierten Lymphknoten gewonnen und in Gegenwart von Concanavalin A kultiviert wurden, wurde nach einer viertägigen inhalativen Exposition von weiblichen BALB/c-Mäusen gegen 10 ml 1,1-Dichlorethen/m^3^ festgestellt. 1,1-Dichlorethen hatte keinen Einfluss auf den Immunglobulin-E-Spiegel im Blut, auf den Einstrom von Entzündungszellen in die Alveolarräume oder auf die Proliferation von Keulenzellen (Ban et al. 2006; IARC 2019).

Die Exposition von je sechs weiblichen BALB/c-Mäusen gegen 10 oder 15 ml 1,1-Dichlorethen/m^3^ führte zu einer statistisch signifikant erhöhten Anzahl von plaquebildenden Zellen in den Lymphknoten der Lunge und bei der höchsten Konzentration auch in Milzzellen. Nur in den Lymphknoten der Lunge wurde eine erhöhte Ausschüttung von Interferon-γ bei der höchsten Konzentration detektiert (Ban et al. 2003).

Die Serumspiegel von TNFα und IL-6 stiegen bei männlichen Swiss-Mäusen nach einmaliger oraler Gabe von 100, 150 oder 200 mg 1,1-Dichlorethen/kg KG nach sechs Stunden an und nahmen dann tendenziell ab. Die maximale Nieren- und Leberschädigung trat 16 bzw. 24 Stunden nach der Behandlung auf. Es bestand eine positive Korrelation zwischen den Schäden am Nierentubulus und der immunsuppressiven Wirkung (antikörperbildende Zellen im Serum und Aktivität der natürlichen Killerzellen vermindert) (Ban et al. 1998).

## 6 Bewertung

Kritischer Effekt ist die kanzerogene Wirkung von 1,1-Dichlorethen in den Nieren und der Schilddrüse von Ratten und in den Nieren, der Leber und der Lunge von Mäusen.

**Krebserzeugende Wirkung. **Nach 2-jähriger inhalativer Exposition erwies sich 1,1-Dichlorethen bei F344-Ratten und B6C3F1-Mäusen ab der niedrigsten Konzentration von 25 bzw. 6,25 ml/m^3^ als kanzerogen. In der Nase traten bei den Ratten erhöhte Inzidenzen von Adenomen des respiratorischen Epithels, bei weiblichen Ratten C-Zelladenome und C-Zellkarzinome der Schilddrüse und bei männlichen Ratten renale Tubuluskarzinome auf. Derartige renale Tubuluskarzinome sind gemäß historischer Kontrolldaten des NTP sehr selten. Bei männlichen Mäusen traten Nierenadenome und -karzinome, bei weiblichen Mäusen hepatozelluläre Karzinome, bronchiolo-alveoläre Karzinome sowie Hämangiosarkome der Leber und Hämangiome und Hämangiosarkome in verschiedenen Organen auf. Die Inzidenz der seltenen Hepatocholangiokarzinome nahm konzentrationsabhängig zu. Der Entstehungsmechanismus der jeweiligen Tumoren ist nicht vollständig erforscht. Es ist zu vermuten, dass dieser in Zusammenhang mit der toxischen Wirkung reaktiver Intermediate im Metabolismus, vor allem von 1,1-Dichlorethenepoxid, steht, die zu Zellschäden, Zellproliferationen und Inflammationen in metabolisch kompetenten Geweben führt (siehe Abschnitt 2). Hierzu ist hervorzuheben, dass das strukturell ähnliche Ethylenoxid von der Kommission in Kanzerogenitäts-Kategorie 2 eingestuft wurde (Greim 2002). Die vorliegenden Daten geben Hinweise auf eine genotoxische Wirkung in Zielgeweben der Kanzerogenität; ob diese oder die Toxizität für die Kanzerogenität im Vordergrund steht, ist derzeit unklar. Somit wird 1,1-Dichlorethen in Kanzerogenitäts-Kategorie 2 umgestuft. Damit entfallen MAK-Wert und Spitzenbegrenzung.

**Keimzellmutagene Wirkung. **1,1-Dichlorethen ist nach metabolischer Aktivierung in vitro mutagen und klastogen. An In-vivo-Tests in metabolisch kompetenten Geweben, die die Zielgewebe der Kanzerogenität darstellen, liegen nur positive Indikatortests vor, die auf eine genotoxische Wirkung hinweisen. Aussagefähigere Tests auf Mikronuklei oder Chromosomenaberrationen in diesen Zielgeweben fehlen. Aufgrund der negativen Ergebnisse im Knochenmark und in validen Studien an Keimzellen ist zu vermuten, dass die reaktiven Metaboliten lokal begrenzt reagieren und die Keimzellen nicht erreichen bzw. dort nicht gebildet werden. Deshalb wird 1,1-Dichlorethen nicht in eine Keimzellmutagenitäts-Kategorie eingestuft.

**Fruchtschädigende Wirkung. **Da kein MAK-Wert abgeleitet wird, entfällt die Zuordnung zu einer risikobasierten Schwangerschaftsgruppe.

In Studien zur pränatalen Entwicklungstoxizität treten entwicklungstoxische Effekte bei Ratten und Kaninchen wie Variationen, Ossifikationsverzögerungen (Ratten) und erhöhte Resorptionen (Kaninchen) nur bei gleichzeitiger Maternaltoxizität in Form von verringerter Körpergewichtszunahme auf (ECHA 2023; Henschler 1979, 1986; Murray et al. 1979). Ein Verdacht auf eine entwicklungstoxische Wirkung (Gruppe B (Verdacht)) lässt sich damit nicht begründen.

**Hautresorption. **Zur Aufnahme von 1,1-Dichlorethen über die Haut liegen In-vitro-Untersuchungen mit Humanhaut vor. Bei Zugrundelegung des hierbei ermittelten Fluxes ergäbe sich für unverdünntes 1,1-Dichlorethen unter Standardbedingungen (2000 cm² Hautfläche, eine Stunde Exposition) eine erhebliche Gesamtaufnahme von 289 mg. Aus Tierversuchen liegen Hinweise auf systemisch kanzerogene Wirkungen nach Inhalation der Substanz vor. Ein Auftreten entsprechender Wirkungen kann auch nach dermaler Aufnahme nicht ausgeschlossen werden. 1,1-Dichlorethen wird daher mit „H“ markiert.

**Sensibilisierende Wirkung. **Zu 1,1-Dichlorethen liegen keine klinischen Daten vor. Ein Local Lymph Node Assay zeigte bis zu einer Konzentration von 50 % ein negatives Testergebnis. Studien mit tierversuchsfreien Alternativverfahren lieferten ebenfalls ausschließlich negative Ergebnisse. 1,1-Dichlorethen wird daher nicht mit „Sh“ markiert. Es gibt keine Hinweise auf eine eigenständige atemwegssensibilisierende Wirkung. Es erfolgt daher keine Markierung mit „Sa“.
